# Rosacea Topical Treatment and Care: From Traditional to New Drug Delivery Systems

**DOI:** 10.1021/acs.molpharmaceut.3c00324

**Published:** 2023-07-21

**Authors:** Ana Cláudia Paiva-Santos, Tatiana Gonçalves, Diana Peixoto, Patrícia C. Pires, K. Velsankar, Niraj Kumar Jha, Vivek P. Chavda, Imran Shair Mohammad, Letícia Caramori Cefali, Priscila Gava Mazzola, Filipa Mascarenhas-Melo, Francisco Veiga

**Affiliations:** † Department of Pharmaceutical Technology, Faculty of Pharmacy of the University of Coimbra, University of Coimbra, Azinhaga Sta. Comba, 3000-548 Coimbra, Portugal; ‡ LAQV, REQUIMTE, Department of Pharmaceutical Technology, Faculty of Pharmacy of the University of Coimbra, University of Coimbra, Azinhaga Sta. Comba, 3000-548 Coimbra, Portugal; § Health Sciences Research Centre (CICS-UBI), University of Beira Interior, Av. Infante D. Henrique, 6200-506 Covilhã, Portugal; ∥ Department of Physics, Sri Sivasubramaniya Nadar College of Engineering, SSN Research Centre, Kalavakkam, Tamil Nadu 603110, India; ⊥ Department of Biotechnology, School of Engineering and Technology, Sharda University, Greater Noida, Uttar Pradesh 201310, India; # Department of Biotechnology, School of Applied and Life Sciences (SALS), Uttaranchal University, Dehradun, Uttarakhand 248007, India; 7 School of Bioengineering and Biosciences, Lovely Professional University, Phagwara, Punjab 144411, India; 8 Department of Biotechnology Engineering and Food Technology, Chandigarh University, Mohali, Punjab 140413, India; 9 Department of Pharmaceutics and Pharmaceutical Technology, L. M. College of Pharmacy, Ahmedabad, Gujarat 380008, India; 10 Department of Radiology, City of Hope Cancer Center, 1500 East Duarte Rd., Duarte, California 91010, USA; 11 Institute of Biology, University of Campinas (UNICAMP), Campinas, São Paolo 13083-862, Brazil; 12 Center for Biological and Health Sciences, Mackenzie Presbyterian University, São Paulo, São Paulo 01302-907, Brazil; 13 Faculty of Pharmaceutical Sciences, University of Campinas (UNICAMP), Campinas, São Paolo13083-871, Brazil

**Keywords:** Rosacea, Skin, Therapy, Cosmetic, Drug Delivery System, Nanoparticle

## Abstract

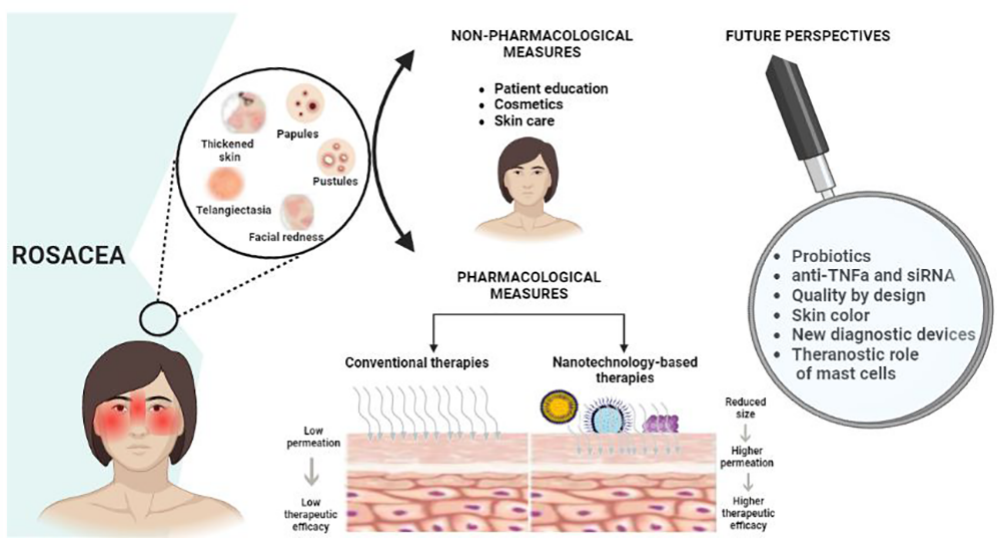

Rosacea is a multifactorial chronic inflammatory dermatosis characterized by flushing, nontransient erythema, papules and pustules, telangiectasia, and phymatous alterations accompanied by itching, burning, or stinging, the pathophysiology of which is not yet fully understood. Conventional topical treatments usually show limited efficacy due to the physical barrier property of the skin that hinders skin penetration of the active ingredients, thereby hampering proper drug skin delivery and the respective therapeutic or cosmetic effects. New advances regarding the physiopathological understanding of the disease and the underlying mechanisms suggest the potential of new active ingredients as promising therapeutic and cosmetic approaches to this dermatosis. Additionally, the development of new drug delivery systems for skin delivery, particularly the potential of nanoparticles for the topical treatment and care of rosacea, has been described. Emphasis has been placed on their reduced nanometric size, which contributes to a significant improvement in the attainment of targeted skin drug delivery. In addition to the exposition of the known pathophysiology, epidemiology, diagnosis, and preventive measures, this Review covers the topical approaches used in the control of rosacea, including skin care, cosmetics, and topical therapies, as well as the future perspectives on these strategies.

## 1 Introduction

Rosacea is a chronic inflammatory dermatosis that affects a small percentage of the world population. Rosacea is considered a chronic skin disorder owing to its prolonged course, periods of exacerbation and remission, and manifestations, including persistent erythema (redness) that resembles sunburn.^1,2^ Although rosacea is not considered life-threatening, it still has profound negative psychological and social effects on the quality of life of patients and presents a high likelihood of depression, social phobia, and anxiety. Depending on the morphological features, they can be classified into four major subgroups: erythematotelangiectatic rosacea (ETR) (subtype 1), papulopustular rosacea (PPR) (subtype 2), phymatous rosacea (subtype 3), and ocular rosacea (subtype 4).^3^

ETR rosacea is a subtype that most people are familiar with, and it typically manifests as persistent redness in the central face region, often accompanied by telangiectasia.^4^ In turn, PPR rosacea is characterized by a variable number of small domed papules and superficial pustules associated with erythema and edema distributed in a centrofacial pattern.^5^ Ocular rosacea presents several nonspecific signs and symptoms, such as eyelid inflammation, photosensitivity, telangiectasias of the eyelid margins, redness of the conjunctiva, tearing, irritation, a sensation of foreign bodies, burning, and stinging; it is often underdiagnosed, and there is no laboratory test for its detection.^5−7^ Late diagnosis can compromise vital vision structures, which can lead to visual impairment.^5^ Ocular rosacea can be classified into three grades according to the signs and symptoms presented: grade one is characterized by mild itching, dryness, telangiectasia, and palpebral erythema; grade two corresponds to a burning sensation, eyelid with erythema and edema, and chalazion; and grade three corresponds to photosensitivity, blurred vision, corneal changes, severe eyelid changes, loss of eyelashes, and severe inflammation of the conjunctiva.^8^ Phymatous rosacea manifests as thickening of the skin with irregular surface contours and nodules as a consequence of several factors, such as fibrosis, sebaceous hyperplasia, and lymphedema.^6^ This condition primarily affects the nose (rhinophyma) but can also affect any facial region that has sebum secretion, such as the chin (gnatophyma), forehead (metophyma), eyelids (blepharophyma), and ears (otophyma).^6^ In dermatologists’ everyday clinical practice, patients may morphologically manifest either one rosacea subtype or a combination of rosacea subtypes and complain of increased sensitivity of facial skin followed by burning, stinging, pain, or pruritus.^2,9^

Considering the plethora of overlapping morphological presentations, several unanswered questions related to rosacea physiopathology remain.^10^ Generally, it is known that inflammatory and vascular effects characteristic of rosacea result from an exacerbated innate immune response and aberrant neurovascular signaling triggered by numerous environmental stimuli and endogenous factors based on a certain genetic background.^7^ On the other hand, as in acne, skin microbiome alterations may be related to the likely pathogenesis of rosacea by instigating or propagating the exacerbated immune response.^11^

The management of rosacea remains a challenge to dermatologists. Treatment options for rosacea may include skin care, systemic or topical therapies, laser- and light-based therapies, invasive methods (e.g., microneedling), and several combinations of these options.^12^ The only Food and Drug Administration (FDA)-approved oral drug is 40 mg modified-release doxycycline once daily for the treatment of inflammatory lesions of PPR.^13^ However, off-label use of numerous drugs is common in the treatment of rosacea.^10^ Oral azithromycin has been used as a therapeutic alternative in patients who present with PPR rosacea and who cannot be prescribed tetracycline.^10^ Minocycline and clarithromycin are two antibiotics that are also commonly prescribed for this condition.^14^ Additionally, drugs from other therapeutic classes have been used to control PPR symptoms (e.g., erythema and flushing with papules and pustules), such as oral contraceptives, amitriptyline, clonidine, pimozide, aspirin, β-blockers, ondansetron, and COX-2 inhibitors.^15^ Highly severe cases of PPR require the prescription of oral isotretinoin.^13^ Other drugs, such as ketoconazole and prednisolone, have also been prescribed for this condition.^10^ However, oral therapy is related to systemic side effects, and bacterial resistance is one of the main concerns associated with the oral administration of antibiotics.^16^

Topical therapy is often an alternative to oral therapy, since it allows local action related to fewer side effects and simultaneously provides greater ease and convenience of application.^17^ FDA-approved topical therapies consists of metronidazole (MTZ) 0.25%, 0.75%, and 1% cream, gel, and lotion; azelaic acid (AZA) 15% gel; sodium sulfacetamide 10%; sulfur 5%; and sodium sulfate 10%. In addition, these other formulations are also used as off-label therapies: topical brimonidine 0.33% gel, 1% oxymetazoline cream, 1% ivermectin cream, calcineurin inhibitors such as 0.1% tacrolimus and 1% pimecrolimus cream, topical retinoids, and 5% permethrin.^17^ These topical formulations have low therapeutic efficacy, which is related to the low permeation of the formulation on the skin.^18^ To overcome this limitation, nanoparticles have been widely studied since, due to their reduced size and character, they manage to have higher skin permeation rates and, consequently, greater therapeutic efficacy because a greater amount of drug is available in the site of action.^18^ These new drug delivery systems have enormous potential in formulations for topical applications since, as the skin is an impenetrable physical barrier to most substances, developing products that can overcome this barrier is an extremely important step for dermatoses.^18^ In the specific case of rosacea, studies carried out using these new drug delivery systems have revealed the promising role they will have in the therapeutic arsenal for the management of chronic inflammatory dermatoses, as these formulations present greater permeation in the skin because their reduced size allows penetration occurs through intracellular and extracellular transport and they have greater cutaneous retention, which in turn reduces the frequency of application of the formulation.^18^

Moreover, skin care plays a vital role in the management of rosacea.^19^ Patients should cleanse the skin morning and evening using gentle cleansing products with a neutral or slightly alkaline pH, preferably syndets, to avoid damaging the skin barrier, preparing the skin for the application of an extremely important moisturizer on this sensitive skin.^19,20^ Considering that the sun is a triggering factor, the application of sunscreen with a sun protection factor (SPF) of 30 or greater containing ultraviolet (UV) B and UVA protection is crucial to avoid possible exacerbation and worsening of the disease.^21^ In addition to these cosmetics, corrective cosmetics to camouflage redness can also be used to minimize the negative psychological effects that the characteristic facial appearance of rosacea has on these patients.^22^

Currently, the effects of probiotics on rosacea management have been studied, since they are associated with an alteration in the skin microbiome.^23^ In addition, studies are currently being carried out to assess the possible benefits of siRNA- and TNF-α-based therapies in the treatment of rosacea. Concerning the diagnosis of rosacea, new techniques have been studied to obtain an early stage diagnosis of rosacea and assess the involvement of the deeper layers of the skin.^24^

It should be noted that patient lifestyle management plays a vital role in rosacea treatment.^25^ Therefore, patients should be aware of the factors that can exacerbate and/or trigger characteristic signs and symptoms of rosacea to avoid them.^26^ For instance, in the current context of the COVID-19 pandemic, instruction regarding the choice of personal protective masks and daily cosmetic skin care is essential, since individual protection measures have a high potential not only to intensify preexisting dermatological conditions but also to initiate new pathological processes.^27^

Overall, the treatment of rosacea must be personalized based on its subtypes, dermatosis severity, quality of life implications, comorbidities, trigger factors, and treatment compliance.^28^ The present paper intends to address recent literature describing the following aspects of rosacea: pathophysiology, epidemiology, diagnostic methods, available topical care and therapies, and innovative treatments.

## 2 Rosacea

### 2.1 Pathophysiology

**Figure 1:**
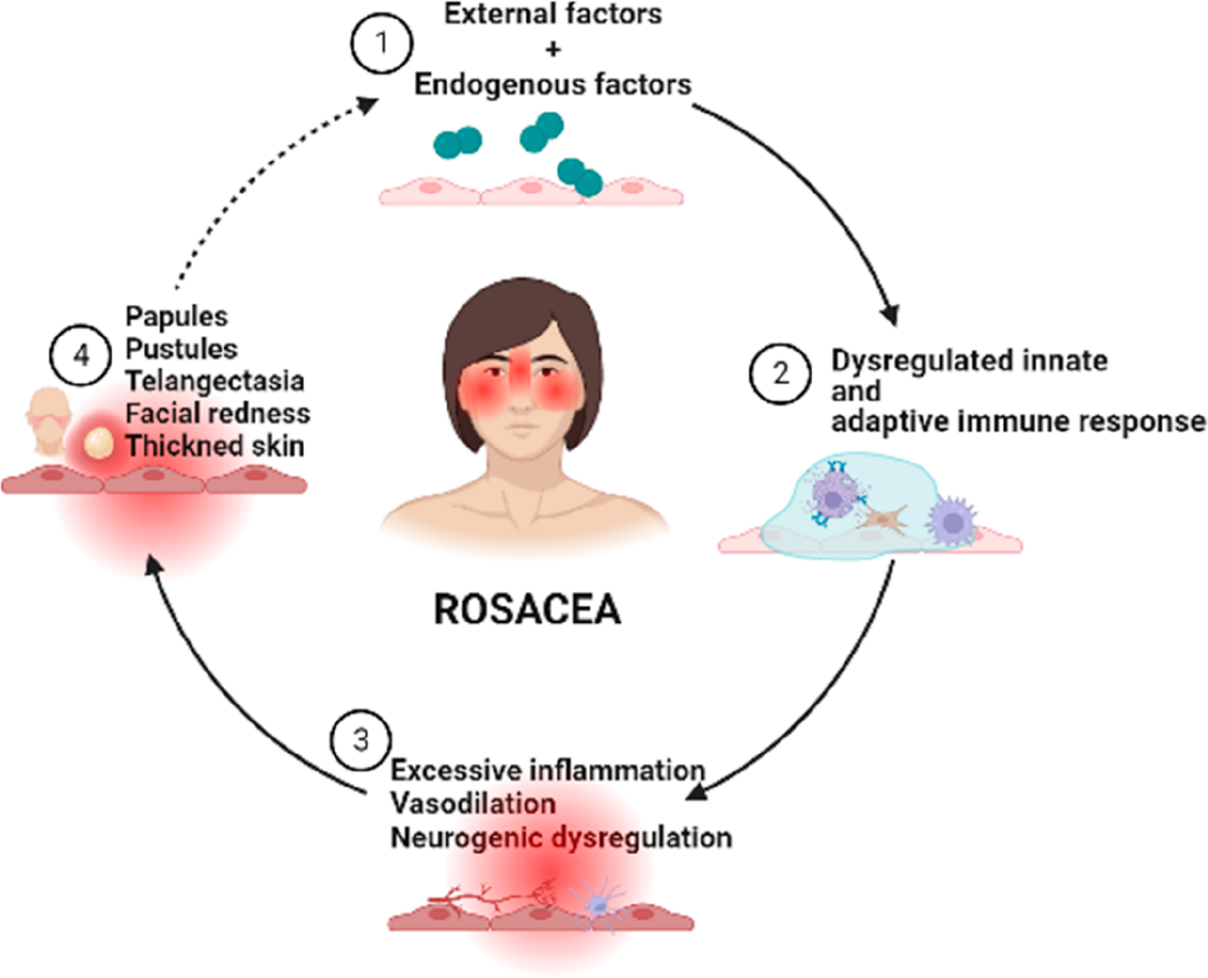
Summary representation of the main aspects involved in the development and manifestation of signals of rosacea (produced with Biorender).

Rosacea is a chronic inflammatory skin condition that mainly affects the face, with a physiopathology related to a multifactorial etiology in genetically predisposed individuals, as happens with other common skin conditions.^4,10,29,30^ In addition, external stimuli are also associated with this dermatitis, since rosacea can also be triggered or worsened by ultraviolet (UV) radiation, diet, high temperatures, skin barrier disruption, psychosocial stress, microbiome, and hormonal alterations.^10^ These external and endogenous factors can lead to a dysregulated innate and adaptive immune response prone to excessive inflammation and vasodilation combined with neurogenic dysregulation.^23^ Collectively, all of the mentioned mediators and processes orchestrate vascular and inflammatory effects that are characteristic of rosacea. Figure 1 schematizes, for better visualization, the main aspects involved in the pathophysiology of rosacea.

Toll-like receptors (TLRs) recognize microbial components, tissue damage, and ultraviolet light-induced apoptotic cells.^31^ External stimuli are driving factors that increase TLR2 activation, one member of the TLR family that is highly expressed in keratinocytes of patients with rosacea. TLR-2 activation triggers a cascade of inflammatory and vasoactive peptides, such as cathelicidins and kallikrein 5, which are also overexpressed in the epidermis of rosacea patients.^32,33^ Kallikrein (KLK) 5 is the main serine protease responsible for cleaving cathelicidin into its active form, the LL-37 peptide.^34,35^ The LL37-induced effects, including chemotaxis of leukocytes, stimulation of angiogenesis, and activation of NF-KB, are collectively correlated with the phenotypic features of rosacea, such as facial erythema, telangiectases, and papules and pustules.^10^ In addition, vascular dysfunction and the release of proinflammatory neuropeptides also occur in rosacea.^10^ Moreover, the activation of T helper (Th) 1, Th17, and B cells promotes inflammation due to the production of interferon-c, IL-17, and immunoglobulins.^23,36^

Recent developments in the field of rosacea have suggested that patients overexpress transient receptor potential (TRP) vanilloid type (TRPV) 1, TRPV4, and TRP ankyrin 1 (TRPA) ion channels commonly found on sensory neurons and keratinocytes. These TRPs are highly stimulated by thermal, chemical, or mechanical factors.^37−39^ Once stimulated, TRPs trigger the cellular release of substance P, pituitary adenylate cyclase-activating polypeptide (PACAP), vasoactive intestinal peptide (VIP), or calcitonin gene-related peptide (CGRP). Substance P is a vasoactive peptide involved in local blood flow regulation and mast cell degranulation inductions, leading to increased levels of proinflammatory cytokines (e.g., interleukin (IL)-1, IL-3, and IL-8), chemokines (e.g., CCL2, CXCL9, CXCL10, CCL5, and CXCL8), and tumor necrosis factor-α (TNF-α).^40,41^ Overall, the findings presented above suggest that neurogenic inflammatory processes are also probably active in rosacea.

Moreover, oxidative stress has a significant role in rosacea, showing that this process can trigger the production of reactive oxygen species (ROS) in the skin. This causes an alteration in the protein, lipids, and neutrophils and an increase in the levels of LL-37, cytokines, and inflammatory mediators, namely, IL-1 and TNF-α.^36,42^ The secretion of cytokines such as TNF-α, IL-1, and IL-6 is due to a change in the permeability of the cutaneous barrier leading to inflammation, which is perhaps connected to an attempt of the epidermis to self-repair.^4^

### 2.2 Epidemiology and Diagnosis

Although rosacea can occur at any age, it is more prevalent in individuals aged over 30 years old.^10,43,44^ In a recent systematic review, the worldwide prevalence of rosacea was estimated to be 5.5% of the adult population. Moreover, men and women were found to be equally affected.^45^ Additionally, rosacea is more prevalent in lighter and sun-sensitive skin tones (Fitzpatrick skin phototypes I–IV).^43^ Yet, it should be emphasized that rosacea is not rare in darker skin tones (Fitzpatrick phototypes V and VI), but it is likely unrecognized and underdiagnosed, since erythema and telangiectasia are more difficult to detect.^46^

As for diagnosis, given that there are no histological or serological indicators, rosacea is diagnosed based on clinical observation.^47^ Rosacea can be classified into four different subtypes according to signals and symptoms:^48^ erythematotelangiectatic (ETR) rosacea, papulopustular (PPR) rosacea, fimatous rosacea, and ocular rosacea.^49^ However, this classification has proven to be limiting, as some patients present symptoms of more than one subtype and there can also be a progression from one subtype to another.^50^ Consequently, this classification has been replaced with one based on phenotypes that focuses on the cutaneous aspects of rosacea, allowing for a personalized clinical evaluation and treatment for each patient.^51^ In patients with rosacea, it is common to obverse telangiectasia and persistent facial erythema, which can worsen periodically in the presence of trigger factors.^43,48^ It is also common to find dome-shaped papules and pustules, and redness can be followed by nodules.^43,48^ In addition to these clinical signs, various symptoms are mentioned by the patients, such as a tinkling burning sensation, especially in the frontal portion of the face.^52^ Localized facial edema as well as dry skin that can be harsh, scaly, and/or with pruritus can also occur.^13^ The diagnosis of rosacea belatedly occurs in people with darker skin tone, thus increasing comorbidity in these populations.^46^

## 3 Prevention

Rosacea has several triggering factors: temperature, emotions (stress and anxiety), food and drink, weather, heavy exercise, and health conditions can have a huge impact on the lives of those who live with this dermatosis.^53^ Some of these exacerbating factors, such as hot temperatures, act directly to trigger vasodilatation, while other factors increase skin inflammation by distinct mechanisms.^53^ Therefore, patient education on nontherapeutic measures is essential, as it can help prevent and control exacerbations.^53^

Dietary adjustments, for example, are often recommended to patients with rosacea, since diet may potentiate rosacea symptomatology.^54^ Thus, suggestions to avoid “exacerbating” foods and drinks are frequent in clinical practice. Interestingly, countless patients describe rosacea exacerbations with spicy foods or with hot drinks.^55^ According to the type of stimulus, dietary factors can be subdivided into four subcategories: heat-related, as it has been described that hot drinks, including hot coffee and hot tea, act as exacerbating factors;^56,57^ alcohol-related, which is another exacerbating factor comprising wine and hard liquor;^58^ capsaicin-related, which is present in some spices and peppers (e.g., cayenne pepper and red pepper); and cinnamaldehyde-related,^53^ a compound that is found in numerous apparently unrelated foods such as tomatoes, citrus, cinnamon, and chocolate.^54^ Heat and capsaicin activate several vanilloid channels (TRPV1–6) that cause vasodilation and inflammation-induced hyperalgesia, resulting in flushing and burning.^54^ Mustard oil and cinnamaldehyde activate TRPA1 receptors, causing flushing symptoms.^54^ Dietary factors are very important and must be taken into account. Currently, an intestine–skin connection has been found, which makes it important for patients with rosacea to be aware of the importance of fiber intake in the sense that fiber contributes to a healthy intestinal microbiome, and some vegetable fibers also play the role of prebiotics.^59^ In addition to consuming a diet rich in fiber, these people may also be advised to take probiotics, since some strains can act on the skin, improving barrier function and decreasing sensitivity.^60^ There are still not enough robust data on the role that some nutrients play in the symptomatic relief of rosacea.^61^ However, some studies are beginning to show that omega 3 fatty acids, namely, eicosapentaenoic acid (EPA) and docosahexaenoic acid (DHA), can competitively inhibit proinflammatory pathways, since they are substrates of anti-inflammatory prostaglandins, and that zinc can have antioxidant and anti-inflammatory actions and is essential for the development of the innate immune system.^55,62^ However, more studies are needed to prove that these specific nutrients alleviate the symptoms of rosacea.^54^

Sun exposure is also a triggering factor for rosacea symptoms and is one of the most common factors for flushing.^21^ The worsening of symptoms caused by UV radiation can be a result of three mechanisms: the promotion of the proinflammatory cascade due to the overexpression of cathelicidin induced by vitamin D; the proliferation of the skin vasculature increased by UVB light; and the increase in ROS and the KLK5-cathelicidin inflammatory cascade due to excess UV radiation.^63^

The other two factors that can worsen rosacea symptoms are alcohol and tobacco.^64^ On the one hand, alcohol triggers transient flushing, accelerating the diesease’s progression^54^ and increasing the risk of developing rosacea;^65^ on the other hand, the nicotine present in tobacco has an angiogenic action and can trigger rosacea symptoms.^64^ A study revealed the highest prevalence of ETR rosacea in active smokers, and this prevalence may be associated with the effects of nicotine.^64^ As these are two substances that can negatively affect the patient’s life, these patients should be alerted to the importance of restricting the intake of alcoholic beverages and should avoid smoking.^64^

In addition to the exacerbating factors mentioned before, other elements contribute negatively to rosacea patients. The extensive use of facial masks (pandemic context) has exacerbated several facial dermatoses,^66,67^ since it provides a warm, humid,^66^ and occlusive^68^ environment leading to dysbiosis of the skin flora.^27,68^ The increase in cutaneous dermatosis was more noticeable in healthcare professionals and other professionals who wore facial protective equipment for many hours.^66,67^ The appearance or aggravation of facial skin diseases as a consequence of the use of masks depends on several factors, such as the type and composition of the mask and previous diagnosis of skin diseases, and it is also related to the length of time the mask has been used.^67^ Polypropylene masks can cause allergic contact dermatitis (ACD) due to the presence of dispersed textile dyes,^68^ elasticity, formaldehyde,^67^ and bronopol.^69^ Patients should be aware of the use of tissue masks, as they cause fewer skin reactions than surgical masks and N95 masks, and should also be advised that surgical masks are disposable and should not be reused.^67^ The choice of fabric masks should focus on fabrics with a lower thread count and a smooth surface with few folds, as they reduce friction between the fabric and the skin, and preferably those in light colors, as the dark colors absorb radiation and, consequently, cause an increase in skin temperature.^68^ Metal nasal bridges and handles can also cause ACD, so masks without metal parts and with adjustable straps should be preferred instead of extensible ones.^68^

## 4 Cosmetics/Skin Care

Chronic inflammation, characteristic of rosacea, can lead to a wide range of signs and symptoms, including flushing, telangiectasia, inflammatory papules and pustules, and ocular manifestations.^25,70^ Initially, rosacea is characterized by a persistent redness that affects the central region of the face, presenting periods of remission and exacerbation.^13^ During rosacea, couperose appears, especially in the nose region. Given that rosacea modifies facial appearance, this dermatosis can present a significant psychological impact and destabilize health-related quality of life.^71−73^ Therefore, it is essential to instruct the patient on a suitable daily skin routine, which is a key strategy to manage dry appearance, dry sensation, and stinging sensation.^74^ The global ROSacea COnsensus (ROSCO) committee suggests adapting rosacea management according to morphological features and highlights that patients should be encourage to acquire a suitable skincare routine.^75^ The ROSCO guidelines suggest that patients’ skincare routine should comprise the application of sunscreen with a sun protection factor (SPF) ≥30 and cleansing of the skin using gentle cleaning agents.^75^ The skincare routine suggested by ROSCO also emphasizes the frequent application of quality moisturizers, which present the ability to repair and maintain the stratum corneum barrier function, enhance skin hydration, and minimize the likelihood of skin irritation. Moreover, corrective redness makeup can also be used.^20^

### 4.1 Facial Cleansing Products

People with rosacea typically complain about “sensitive skin” as a consequence of either impairment in the epithelial barrier function or its exacerbations. Thus, patients are strongly encouraged to use gentle cleansing products without lipids to avoid further skin barrier damage.^76^ In this pathology, cleansing fluid formulations are the most recommended, since they can be removed without water. For instance, syndets, also known as synthetic detergents, or dermatologic pains are less irritating, as they can minimize dryness in the skin and present a neutral or slightly acidic pH (from 5.5 to 7). These agents are more compatible with the natural acidity of the skin and should be applied on the face using circular movements and then removed with cotton.^19,20,44^ When the use of cleansing products requires rinsing, the skin should be carefully rinsed with warm water to ensure that all the cleansing product is removed and subsequently dried using a soft towel.^76^ These products should not contain surfactants, such as sodium lauryl sulfate (SLS), as they can irritate the skin.^19^ Additionally, patients with rosacea should be encouraged to avoid chemical or physical exfoliants and alcohol-based topical products, since they can cause flushing by exerting an abrasive action.^77^

Decorative cosmetics remotion in rosacea patients should be conducted using skin cleansers that are low-foaming and free of lipids and volatile solvents, which are responsible for worsening facial redness due to their harmful effect on intercellular lipids.^79^ Another possible alternative is cleansing creams, which can be particularly useful in patients with dry rosacea who need complete removal of decorative cosmetics, as these creams provide both cosmetic removal and gentle cleansing of the skin.^79^

Currently, dry or moist facial wipes can be used alternatively to mild cleansing agents and cleansing creams, and dry wipes must be wetted before use.^79^ These cleansing cloths contain a cleansing agent, which is usually a syndet, and the cloth itself allows the skin to be washed.^79^ However, if people with rosacea choose these cloths, they should be advised to choose open weave cloths, as cleansing of sensitive skin needs to be less aggressive to prevent facial redness.^79^ There are also cloths with a moisturizing and cleansing effect simultaneously, as the textured side contains the cleansing product and the smooth side contains the moisturizer.^80^ Cleansing bags are another variant of these towels, where there are two cleansing cloths separated by a porous membrane that controls the release of ingredients on the skin surface, but patients with rosacea should be careful when using this product, as it often contains botanical products that may be contraindicated in this population.^79^ After cleansing the skin, micronized thermal water can be applied due to its calming effect.^20^

### 4.2 Facial Hydration

#### 4.2.1 Hydration

Rosacea is a skin condition characterized by a damaged cutaneous barrier that leads to increased transepidermal water loss (TEWL).^13,76^ Therefore, skin hydration plays a key role in creating a favorable environment that promotes the repair of the skin barrier.^25^ Moisturizers promote the repair of the skin barrier, as they tend to reproduce the effect performed by sebum and the intercellular lipid compounds of sphingolipids, which occur naturally in the skin.^79^

Due to skin sensibility, these patients should avoid using moisturizers that contain vegetables and/or animal oil, as this can create a medium ideal for the growth of bacteria.^79^ The moisturizers that have shown more efficacy in the prevention of exacerbations are formulations that contain occlusives and humectants; since silicones are inert ingredients with a high moisturizing capacity, formulations containing silicone are often the moisturizing products of choice in this skin pathology.^80^ In this group of patients, preference should be given to formulations with a low lipid content and avoiding O/W creams, which require surfactants that can lead to hypersensitivity.^19^ In the formulation of these cosmetics, the use of fragrances and/or alcohol should be avoided due to the potential of causing hypersensitivity.^25^

#### 4.2.2 Hydration with Additional Cosmetic Actives and Functions

The increased relevance of cosmetics in daily skin care for patients with rosacea has led to the development of cosmetic products where the active ingredients have a primary objective of having a calming, anti-inflammatory effect and/or promote the stabilization of blood vessels as their core property.^76,81^ Within this, it is fundamental to limit formulations that have active ingredients with antiaging properties, as these can have an exfoliating effect and cause irritation; formulations that increase blood flow and, similar to other cosmetics products, the use of formulations containing essential oils, menthol, and camphor should be avoided.^82^

Currently, some botanical ingredients are added to the formulation for rosacea, such as leaves of *Ginkgo biloba*, *Camellia sinensis*, *Aloe vera* mucilages, *Alantoína*, *Matricaria recutita*, and *Glycyrrhiza inflata*.^76^ All these botanical ingredients share an anti-inflammatory property, although the mechanism of action varies from one ingredient to another.^76^ In addition to this anti-inflammatory property, *Ginkgo biloba* and *Matricaria recutita* modify the cutaneous microcirculation, which, in conjunction with the anti-inflammatory effect, reduces erythema.^76^

Maggioni et al. conducted a cohort study with 20 patients with rosacea to assess the effect that BK46 serum has on skin barrier function and on the reduction of rosacea symptomatology, namely, in terms of TEWL, capillary diameter, index of erythema, redness, and telangiectasia.^83^ The formulation of BK46 serum consists of a combination of various cosmetic ingredients, such as potassium azeloyl diglycinate, squalene, dipotassium glycyrrhizate, *Aloe barbadensis* leaf juice, sodium hyaluronate, 6-cross polyacrylate, and xanthan gum, which act synergistically with each other, allowing a reduction in rosacea symptoms.^83,84^ Azeloyl diglycinate has a moisturizing action that is important to restoring skin balance;^84^ squalene has an emollient action;^85^ dipotassium glycyrrhizate acts as a skin conditioner and emollient;^86^ xanthan gum has a film-forming action;^87^ hyaluronic acid is a skin moisturizer and conditioner;^88^ polyacrylate-6 cross polymer performs a moisturizing action and helps to re-establish the skin’s barrier function, preventing water loss; and the juice of the aloe leaves has moisturizing, emollient, and film-forming properties.^89^ Patients included in the study were instructed not to change their daily routine and to apply test samples in the morning and evening after skin cleansing for 56 consecutive days.^83^ The evaluation of the effects of the serum consisted of an instrumental evaluation of skin hydration with a corneometer, evaluation of TEWL using a tewameter, evaluation of the erythema index using an MX18 mexameter, measurement of capillary blood diameter using VIDEOCAP, and a clinical evaluation of redness and telangiectasia.^83^ Instrumental evaluation was carried out at the beginning and after 24 h, 14 days, 28 days, and 56 days, and clinical evaluation was carried out at the beginning of the study and after 14 days, 28 days, and 56 days.^83^ The results obtained in the study showed that the serum forms a protective film on the skin that reinforces skin barrier function, which in turn decreases TEWL, increases skin hydration, and exhibits to a reduction in erythema and visible telangiectasias.^83^ In addition to these effects, patients reported a significant decrease in the sensation of skin dryness and imperfections and considered the formulation to be well tolerated.^83^

### 4.3 Sunscreen

Solar radiation is a triggering factor for an exacerbation of rosacea, so the use of sunscreen is imperative.^19,63^ According to ROSCO recommendations, patients should use SPF ≥ 30, containing ultraviolet (UV)B and UVA filters.^29^ In addition, it is also essential to select formulations that contain a high water content and a low lipid content, as well as advise patients to apply sunscreen frequently, as these hydrophilic formulations are less resistant to water and sweat.^90^ There is still no solid basis on the type of solar filters that should be used;^91^ however, this niche of patients has reported a discomforting sensation of heat in the skin due to organic filters absorbing solar radiation, giving preference to mineral filters, as these do not absorb the solar radiation and do not cause discomfort.^91^

### 4.4 Decorative Cosmetics

Rosacea is a skin condition that can cause redness on the face; therefore, the use of cosmetic products that reduce this redness is important psychologically for the patient, as it camouflages the redness, making the face more harmonious.^22,76^ These cosmetics should not contain any fragrances and should not be irritants or sensitizers due to the low tolerance of skin with rosacea.^77^

After cleaning and moisturizing the skin, cosmetics with color can be applied to mask the redness.^92^ For this effect, green-colored cosmetics are typically used since they help to neutralize redness.^92^ Last, the green-pigmented cosmetic powder can be applied if the patient does not intend to use the foundation. If the patient intends to use a foundation, then green cosmetics should be used, as this will promote the camouflage effect to minimize redness.^92^ The application of cosmetics is particularly useful for patients who do not present severe redness. For those with severe redness where the application of cosmetic powder or foundation is insufficient to cover the redness, the application of vasoconstrictors before the use of cosmetics is necessary.^19^ When the use of vasoconstrictors is not appropriate, then an opaque foundation should be used.^19^ The use of cosmetic foundations allows camouflage of the skin imperfections and erythema characteristic of rosacea, but it is often necessary to use makeup removers that can damage the skin barrier.^80^ In this sense, Tang et al. formulated a cosmetic base based on hemp/cellulose nanocrystals (CNCs) to reduce skin damage caused by facial cleansing products, since this formulation has easy cleaning properties conferred by the effect of cellulose adsorption.^93^ CNCs are biodegradable, biocompatible, have a large surface area and low density, and have excellent colloidal stability.^94^ However, CNCs have strong intermolecular hydrogen bonds, which make it difficult to dissolve CNCs in oily solvents.^94^ Thus, in this formulation, the hydroxyl groups were replaced by polylactic acid (PLA), which is nontoxic, biocompatible, biodegradable, bioabsorbable, has good mechanical strength, and is easy to obtain, giving rise to hemp/CNC-*g*-PLA to improve its aqueous and oily dispersion and thus providing easy cleaning properties.^95^ The researchers evaluated its *in vitro* penetration in a pig skin model using fluorescein isothiocyanate.^93^ The results of this study showed that the hemp/CNC-*g*-PLA cosmetic foundation remained mainly on the stratum corneum surface, indicating that the formulation does not penetrate the skin.^93^ The *in vivo* effectiveness of the hemp/CNC-*g*-PLA cosmetic foundation was evaluated through skin adhesion and corrective properties.^93^ These studies revealed that the formulation has a satisfactory adhesion capacity and improves the pigmentation and redness of the skin.^93^ Additionally, the enormous potential of this type of formulation in patients with facial dermatoses is highlighted.^93^

## 5 Botulinum Toxin

Botulinum toxin (BoNT) is constituted by a light chain and a heavy chain wherein the heavy chain binds to the cholinergic nerve terminal and the light chain inhibits the release of acetylcholine from presynaptic vesicles.^96^ BoNT also has an action on epidermal keratinocytes, macrophages, and mast cells, among others.^97^ BoNT blocks mast cells, providing a reduction in LL-37-induced erythema, and mast cell degranulation suggests a decrease in LL-37-triggered inflammation.^97^ In addition, inhibition of the acetylcholine signaling pathway provides symptomatic relief from facial flushing.^97^ Bloom et al. conducted a pilot study to study the impact of abobotulinum toxin A on erythema and persistent redness in people with ETR rosacea, with abobotulinum toxin A being administered through an intradermal injection.^98^ The choice of abobotulinum toxin A consisted of its greater diffusion and migration, and these characteristics are important in its use in more extensive areas.^98^ This study showed that intradermal injection of abobotulinum toxin A was effective, as it reduced erythema and was safe, as it did not worsen erythema in any patient.^98^ It also revealed that an increase in dose does not convey better results.^98^ In this study, intradermal injection of abobotulinum A has shown promise, but further studies are needed to determine the optimal dose and duration of the toxin for the treatment of erythema and flushing associated with rosacea.^98^ However, since BoNT has a high molecular weight, ablation of the stratum corneum is necessary for BoNT to penetrate the skin.^100^ Thus, the authors performed a retrospective review to assess the safety and efficacy of a nonlaser thermal resurfacing system in the treatment of erythema and flushing refractory to other treatments.^100^ The device used was the Tixel device, which is a thermomechanical system that transfers energy to the skin, causing dehydration of the SC that originates micropores and thus increasing the permeability of the skin.^100^ The association of the Tixel device with sonophoresis increases the topical absorption, as it allows the deposition of the actives in the deeper layers of the skin; that is, in this study, initially, there was a thermal decomposition of the SC followed by an immediate administration of BoNT.^100^ This thermomechanoablative system significantly improved flushing, erythema, and inflammation of the skin, and the side effects were self-limiting and allowed for a homogeneous distribution of botulinum toxin in the skin.^100^ Thus, this method has proven to be effective and safe in patients who present facial erythema due to persistent rosacea.^100^ This method has shown promise, but further studies are needed, as it was carried out with a small number of people, in a short follow-up period, and without a control group.^100^ Niami et al. evaluated the treatment with pulsed dye laser followed by intradermal botulinum toxin type A (BoNTA) in the treatment of erythema and flushing associated with rosacea.^100^ In this study, there was a reduction in erythema, telangiectasias, flushing, and itching, with this treatment having few side effects.^100^ After treatment, patients were followed for three and nine months, and a sustained decrease in erythema was observed, with some patients having a relapse of flushing but less severe than before treatment.^100^ The synergism between pulsed dye laser followed by intradermal BoNTA decreases vascular and neurogenic inflammation.^100^

## 6 Topical Therapies

### 6.1 Metronidazole

MTZ is a nitroimidazole derivative with antibacterial and anti-inflammatory properties. The mechanism of action of topical MTZ has yet to be completely understood, although its effects are considered to result from the decrease in the generation of ROS.^25^ Topical MTZ for the treatment of rosacea consists of a 1% MTZ cream once a day or a 0.75% MTZ cream twice a day.^101^ The topical application of MTZ is considered to be effective in the treatment of moderate to severe rosacea, since it can decrease erythema, papules, and pustules, and when treatment is discontinued the remission of the disease is still verified.^102^ The decrease in papules and pustules, as well as the decrease in erythema, makes MTZ effective in the treatment of PPR rosacea.^103^ Although the application of MTZ is considered safe and tolerable by most patients, adverse effects can occur.^104^ The most common adverse effects after applying the MTZ cream are irritation and dermatitis.^104^

**Figure 2:**
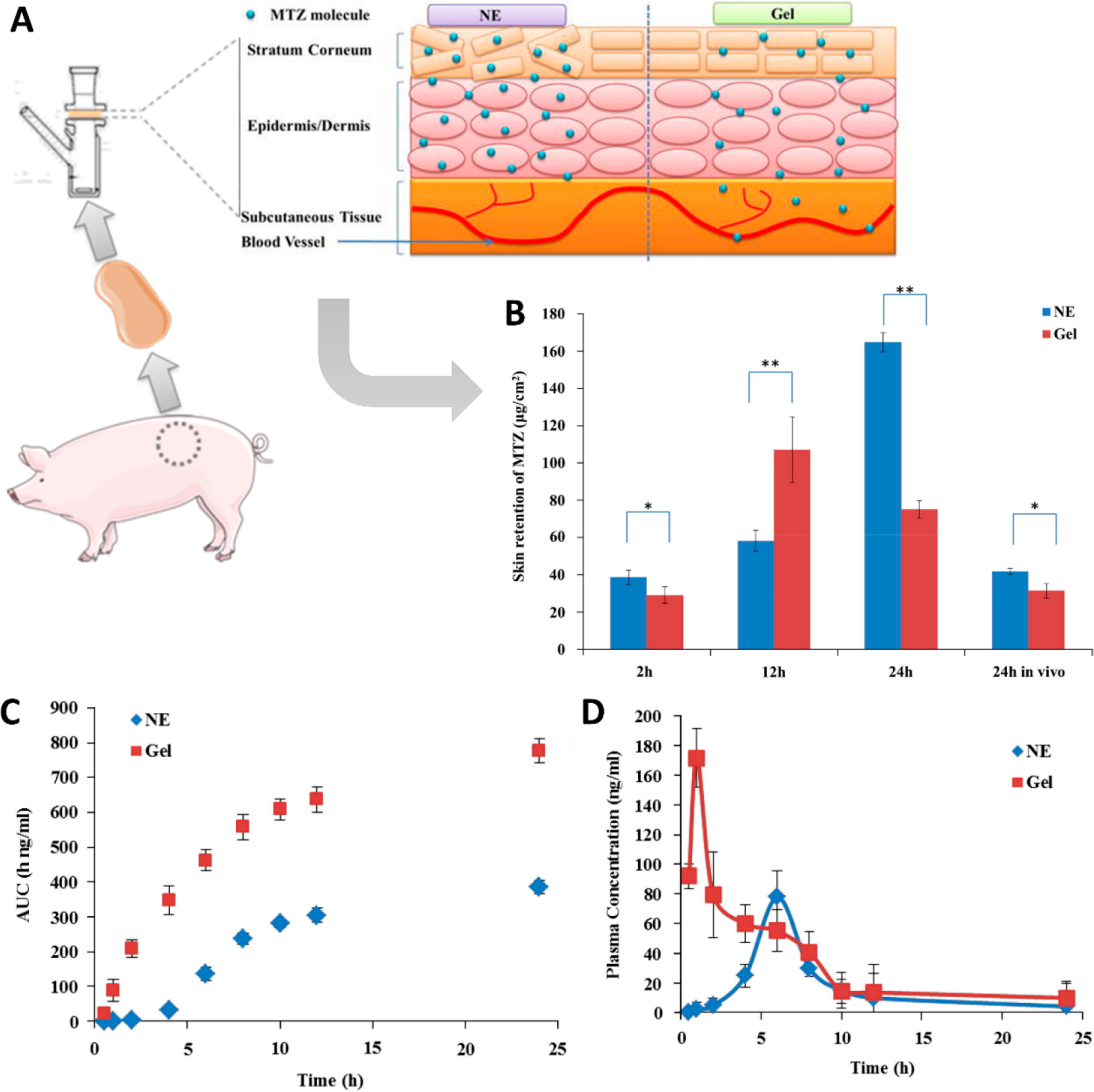
(A) Schematization of MTZ *in vitro* permeation studies through pig skin. (B) Comparison of the amount of MTZ retained in the skin when applied at a dose of 1 mg through NNE and commercial gel. (C) AUC–time profiles and (D) plasma concentration of MTZ after administration via NNE and commercial gel. Adapted from Yu et al.^105^ with permission from Elsevier. Copyright 2014 Elsevier B.V.

Yu et al. prepared various MTZ nanoemulsions (NNEs) to assess the potential of this formulation in topical therapy of rosacea. In this study, the NNE formulation involves Labrafil M1944CS as an oil phase, Cremophor EL as a surfactant, and tetraethylene glycol as a cosurfactant.^105^ The drug was added to the mixture of surfactant and cosurfactant in a proportion (w/w) of 2:1 (Smix), and the NNE obtained was a clear and isotropic solution (Figure 2).^105^ Skin penetration of the drug depends on the water content in the formulation, so the researchers formulated three NNEs whose water contents were 20%, 60%, and 80% w/w.^105^ These studies demonstrated that skin retention was the greatest in the formulation with the highest water content(80% w/w), in other words, NNE O/W.^105^ Moreover, it also demonstrated that the greater the droplet size is, the smaller the cumulative effect of MTZ on the skin, and the increasing the viscosity increases the probability of particle aggregation.^105^ According to that information, an optimized formulation of NNE O/W is clear and transparent, with a low viscosity to facilitate diffusion of the drug to attain a higher therapeutic efficacy, and the droplets should be a round shape.^105^ The droplet size should be between 20 and 30 nm, but after drug incorporation the droplet size increases, since MTZ is a hydrophilic drug and dissolves predominantly in the external aqueous phase.^105^ NNE characteristics were achieved through the NNE formulation with 4.13% Labrafil, 16.42% Smix, and 79.45% water.^105^ To compare drug delivery from NNE with commercial gel, *in vitro* release studies have been carried out.^105^ These results showed that the drug penetration profile in the skin was similar between the NNE and the commercial gel.^105^ At an early stage, the greatest drug retention was obtained by the commercial gel, while after 24 h drug retention in the skin provided by NNE was superior to the drug retention provided by the commercial gel^105^ (Figure 2a). This increase can be explained by the skin reservoir system, which provides a continuous therapeutic effect. In addition to *in vitro* studies, *in vivo* studies have also been carried out.^105^*In vivo* studies found that the gel has a burst effect (sudden and unexpected), and NNE provided greater cutaneous retention of MTZ and lower blood concentration, therefore demonstrating a higher safety profile^105^ (Figures 2b–d). Furthermore, NNE showed the ability to increase MTZ penetration in the skin compared to the gel, since NNE allowed extracellular and intracellular transport, while the gel only allows extracellular transport.^105^ Consequently, NNE provides intra- and extracellular drug localization. NNE has also enabled controlled drug release and a broader distribution of MTZ in the skin, since during the oil phase the surfactant and cosurfactant can interact with epidermal lipids and facilitate diffusion or increase the solubility of MTZ in the skin.^105^ According to these results, NNE and MTZ can be useful alternatives in the treatment of rosacea, and NNE O/W can also serve as a vehicle for hydrophilic drugs in topical therapy.^105^

Shinde et al. formulated the MTZ NLC intending to increase the deposition and retention of hydrophilic MTZ on the skin.^106^ The MTZ NLC was formulated using glyceryl monostearate as a solid lipid, glyceryl monocaprylate as a liquid lipid, Tween 80 as a surfactant, Kolliphor EH 40 as a cosurfactant, Carbopol 974P as a gelling agent, and sodium lauryl sulfate (SLS) as a charge inducer.^106^ Glyceryl monostearate as a solid lipid has two hydroxyl groups and one ester group in its structure that make the molecule more polar, thus allowing hydrogen bonds to form between the hydrogen of the hydroxyl groups of glyceryl monostearate and the oxygen of the nitro group of MTZ as well as between the oxygen of the carbonyl group of glyceryl monostearate and the hydrogen of the hydroxyl group of MTZ.^106^ The choice of glycerol monocaprylate as a liquid lipid falls to the presence of two hydroxyl groups and one ester group in the structure, which promote an increase in the solubilization capacity of MTZ.^106^ Different proportions of solid lipids/liquid lipids were studied, and the optimal ratio of solid lipids/liquid lipids was 8:2, since it allows the maximum incorporation of liquid lipids and, in turn, the maximum drug incorporation.^106^ MTZ NLCs exhibited spherical particles with a uniform size distribution.^106^ The *in vitro* release of MTZ from NLCs shows a biphasic effect, where there is a burst effect at an early stage as a result of the adsorption of drug molecules on the surface of the NLCs and with the increase in the amount of drug in the surface layers of the NLCs, resulting from the cooling of the NLCs from an elevated temperature to room temperature. This effect is important to provide a high drug concentration after application to reach the minimum inhibitory concentration (MIC) and, subsequently, to achieve a sustained release of MTZ from NLCs, which is critical to ensuring the necessary drug levels in the skin for a long period of time. The *in vitro* release of MTZ from NLCs was compared to the *in vitro* release of MTZ from the commercial gel (Metrogyl), and it was concluded that NLCs have more advantages than Metrogyl for rosacea treatment, since the application of NLCs decreases the frequency of application or increases the time interval between each application due to the sustained release, which culminates in greater compliance.^106^ MTZ NLCs also have good spreadability.^106^*Ex vivo* permeation studies have shown that NLCs have greater deposition and drug retention on the skin than commercial gels and cause less systemic exposure than that provided by commercial gels; that is, NLCs present greater safety.^106^ NLCs presented the lowest MIC, showing that lipid encapsulation of the drug increased antibacterial activity, since the reduced size of NLCs and their lipophilic nature facilitates the entry of this transporter through the bacterial wall and they will therefore release the drug directly upon local action.^106^ Through this study, it can be concluded that incorporating MTZ in an NLC increases the therapeutic effect, thereby making it a promising method in the treatment of rosacea.^106^

### 6.2 α-2 Adrenergic agonist

#### 6.2.1 Brimonidine

Brimonidine is an α-2 highly selective agonist^12^ that has a vasoconstrictor effect on small arteries and veins.^14,107^

Topical brimonidine consists of a 0.33% brimonidine gel that should be applied every 12 h when the patient presents erythema.^107^ The application of this formulation has a faster onset of action and sustained action over time, and 0.33% brimonidine gel was the first approved treatment for persistent rosacea facial redness.^108,109^ The vasoconstrictor effect of brimonidine results not only in a decrease in erythema but also in telangiectasias smoothing.^109^ Moreover, brimonidine is related to an anti-inflammatory action,^107^ since the application of brimonidine gel decreases the number of inflammatory cells, mainly mast cells.^110^ The topical application of the gel has a rapid effect on erythema and telangiectasias.^25^ The adverse effects after applying the brimonidine gel are generally tenuous and transient, and the most common adverse effects are redness and worsening of the erythema, as well as redness in the skin surrounding the application site.^12,108,109^ In addition to these effects, brimonidine gel can also cause a lightening effect on the skin due to its vasoconstrictor effect. Since 0.33% brimonidine gel showed minor side effects, it has been proven to be effective and safe in the long term.^12^ However, some patients had a rebound effect during clinical trials,^109^ which may be associated with higher concentrations of drug in the skin due to the alteration of the skin’s barrier function and reduced SC hydration.^111^ Brimonidine gel also showed safety and good long-term tolerability, even when administered concomitantly with other drugs intended for the symptomatic treatment of rosacea.^108,109^

Cationic supramolecular hydrogels formulated with low-molecular-weight gelators (LMWGs) are a promising approach for the topical administration of drugs, as LMWGs organize themselves to form fibers that give rise to thermoreactive supramolecular gels and have a greater biodegradability than polymers.^112^ The formulation of cationic supramolecular hydrogels using bisimidazolium salts (1·2Br), which act as LMWGs, as a cationic surfactant allows the incorporation of anionic drugs, where the positive charge of the surfactant and the negative charge of the drug interact and allow the formation of micelles.^112^ However, 1·2Br allows the formation of gels in the presence of neutral or cationic drugs, providing a quick and effective release, since there are no electrostatic forces between the drug and 1·2Br.^112,113^ Limón et al. proposed a formulation of brimonidine tartrate cationic supramolecular hydrogels.^114^ For the formulation, the researchers used brimonidine tartrate and 1·2Br as LMWGs, ethanol to completely dissolve the 1·2Br, and water as an antisolvent.^114^ The amount of ethanol in the formulation is crucial for the dissolution of 1·2Br and, consequently, for the formation and stability of the gel, and studies have shown that the ethanol content must be between 35% and 50%.^114^ The concentration of 1·2Br must be at least 4 mg/mL for gel formation to be possible at room temperature.^114^ The increase in the concentration of 1·2Br in the 5, 7.5, and 10 mg/mL formulations led to smaller time intervals necessary for gel formation.^114^ However, this increase did not result in any macroscopically significant change in the consistency or appearance of the gel, and the formulation was made with 5 mg/mL 1·2Br.^114^ Gels formulated with these amounts of ethanol, 1·2Br, and brimonidine tartrate remained stable for at least 6 months at 30 °C.^114^ Optimal formulation conditions were chosen and consisted of 5 mg/mL 1·2Br with an ethanol/water ratio of 1:1.^114^ Gel preparation consisted of the addition of brimonidine tartrate dissolved in water to a solution of 1·2Br in ethanol and was carried out at room temperature, since at temperatures above 35 °C there was no gelling and at temperatures below 5 °C there was slight flocculation.^114^ Studies of the formed gel showed that brimonidine tartrate was incorporated both in the fibers and in the interstitial space and revealed that this type of system has a great capacity for drug incorporation, protecting it and promoting its penetration into the skin, thus creating a promising system.^114^ Drug release from the hydrogel was assessed using Franz cells at 32 °C (normal skin temperature), and the results showed a large release of the drug from the hydrogel and that the hydrogel that did not decrease skin penetration.^114^ To evaluate skin permeation, a commercial brimonidine formulation, Mirvaso S, was used.^114^ Brimonidine hydrogel presented slower permeation than the commercial formulation during the first 25 h, the moment when brimonidine hydrogel presents a higher drug flow, or in other words, the hydrogel presents the highest lag time, but this factor was not considered critical in rosacea treatment, since this is a chronic disease.^114^ In addition to permeation on the skin, researchers have also assessed drug retention on skin.^114^ The brimonidine hydrogel provides 3× greater skin retention than a commercial product.^114^ Hydrogel application increased the amount of drug in pharmacological receptors and promoted better skin drug retention, which allows the skin to function as a drug reservoir, thus decreasing the frequency of use.^114^ The researchers assessed the pharmacological efficacy *in vivo*, which demonstrated that the brimonidine hydrogel had greater effectiveness, as it reduced erythema in a short time.^114^

#### 6.2.2 Oxymetazoline

Oxymetazoline is an α-adrenergic receptor agonist drug with high selectivity for the α1-adrenergic receptor and partial selectivity for the α2-adrenergic receptor.^107,115^ The commercially available topical formulation of oxymetazoline consists of a 1% oxymetazoline cream, which is approved to decrease persistent facial erythema associated with rosacea.^107^ The pharmacological action of this formulation is related to its vasoconstrictor effect, which results in a decrease in erythema and visible telangiectasias.^107,117^ Furthermore, it also inhibits neutrophil phagocytosis and oxidative stress, decreasing proinflammatory cytokine production, which helps to reduce inflammation.^117^ The most common adverse effects after applying the oxymetazoline cream are dermatitis at the application site, increased inflammatory lesions, pain, itching, erythema, and paresthesia.^16^ It should be noted that the application of oxymetazoline cream is considered safe and well tolerated.^16^

### 6.3 Azelaic Acid

AZA is a dicarboxylic acid that presents anti-inflammatory, antioxidant, and antimicrobial activities, as well as mild antikeratinizing effects.^12^ AZA naturally occurs in humans and therefore has no mutagenic or teratogenic potential, thus presenting a low risk when used by pregnant women.^12^ Currently, two topical formulations of AZA are available (gel at 15% and cream at 20%) and are used in the treatment of rosacea.^118^ The pharmacological action of AZA on rosacea is based on reducing the expression of cathelicidin and kallikrein, which in turn decreases inflammation.^115^ The daily topical application of these formulations improves symptoms of rosacea because they decrease erythema as well as the number of inflammatory lesions,^118^ having particular effectiveness in the treatment of PPR rosacea.^12^ However, this formulation is not effective in telangiectasias.^25^ The most common adverse effects are irritation, xerosis, and burning; however, the overall treatment is well tolerated by patients, since these adverse effects are transient and, in most cases, mild to moderate.^119^

Dall’Oglio et al. conducted an eight-week, open-label, prospective, multicenter clinical trial with patients with mild to moderate inflammatory rosacea.^120^ In this study, a formulation composed of 15% cream AZA with antioxidant, anti-inflammatory, and antimicrobial activities and 1% dihydroavenanthramide D, which presents anti-inflammatory action and anti-itching, was tested.^120^ The clinical trial was performed for eight weeks, during which the participants applied the cream twice a day, and the clinical evaluation was carried out at week 0 and week 8.^120^ Efficacy was evaluated using the Investigatos Global Assessment (IGA) score and counting the number of inflammatory lesions. The evaluation of erythema was carried out by erythema-directed digital photography (EDDP), and tolerability was evaluated through a self-administered questionnaire.^120^ Before study participants’ inclusion, they underwent a washout period of two or four weeks for topical or oral agents, respectively, and mild cleansers, SPF 50+ sunscreen, and decorative makeup were provided upon entry into the study.^120^ The results obtained in the study showed that there was a significant reduction in the IGA score, EDDP, and inflammatory lesion count.^120^ Only one participant had serious side effects, and tolerability was rated excellent by the majority of participants, showing that this formulation was effective, safe, and well-tolerated.^120^

Tyring et al. performed a clinical trial to evaluate the advantages that AZA foam provides in the sensation of application during treatment in patients who present moderate or severe PPR rosacea with inflammatory lesions and persistent erythema and may or may not have telangiectasias.^121^ Patients who were treated with AZA foam two times a day reported excellent or good tolerability and good cosmetic acceptability.^121^ The side effects of this formulation consisted of pruritus, xerosis, and pain at the application site.^121^ The use of these foams had a high success rate, reducing the number of inflammatory lesions and consequently increasing the quality of life.^121^ This related improvement in the quality of life can be due to the vehicle used in the formulation, since the foam formulation has an easy application and therefore reduces the application time and leaves minimal residue on the skin.^121^ In addition, cosmetic acceptability also increases the therapeutic response, as it leads to greater therapeutic adherence.^121^

AZA is a diprotic acid that at 25 °C has limited water solubility, and this characteristic conditions the topic formulation, since it restricts the vehicles that could be used and the quantity of drug that can be produced.^123^ The topical formulation can occur as a gel or cream where AZA is present as a suspended solid, which limits skin penetration.^123^ Due to the low solubility and low cutaneous penetration, new techniques have been developed for topical AZA formulations.^123^ Tomic et al. proposed *in situ* hydrogel formation with AZA nanocrystals to increase the effectiveness of topical azelaic acid therapy because nanocrystals present greater aqueous solubility and more dissolution of other larger crystals.^123^ Hydrogels are beneficial in formulations with nanocrystals, as they can only be formulated with water.^123^ In addition to greater yielding and cutaneous penetration, which is obtained with nanocarriers, *in situ* hydrogel formation offers countless advantages, such as improved local action of the formulation, and its main advantage is that the *in situ* formulation can be applied as a solution that, once subjected to physiological conditions, turns into a gel, which in turn will have longer retention time and more local drug efficacy because the transformation of the solution into a hydrogel prevents fast elimination.^123^ In this study, researchers evaluated an *in situ* hydrogel formulation consisting of Pluronic F127 and hyaluronic acid-containing AZA nanocrystals obtained by wet media milling technology and subsequently converted into AZA solid nanocrystals by freeze-drying. In this study, researchers prepared six samples in which the AZA concentration remained constant (2% w/w) and the the polysorbate 60 concentration was varied, where the ideal AZA nanocrystal formulation consists of 2% (w/w) and 0.3% (w/w) polysorbate 60.^123^ The intrinsic dissolution rate (IDR) test was conducted at pH 2 and 32 °C to mimic skin temperature, where the IDR of the lyophilizate of AZA nanocrystal suspensions (LNS-AZA) was greater than that of the pure drug. This increase is associated with the increased wettability, AZA solubility, surface energy obtained by nanonization.^123^ An *in vitro* release study demonstrated that AZA release through Pluronic F127/hyaluronic acid hydrogels loaded with the lyophilizate of AZA nanocrystal suspensions (LNS-AZA-PHA) is greater than AZA release through commercial cream.^123^ In addition to these parameters, hydrogel rheology also significantly affects therapeutic efficacy, since it can interfere with spreading and residence time on the skin surface for a topical application.^123^ Being an *in situ* hydrogel formulation, the sol–gel transition temperature (*T*_gel_) is also relevant, and in the LNS-AZA-PHA formulation the gelation can be explained by hyaluronic acid chains bonding to the Pluronic F127 micelles to form an interconnected micelle network (15% Pluronic F127 and 1% hyaluronic acid).^123^ To assess *in vivo* skin absorption, a tape stripping method was used, which showed that the LNS-AZA-PHA formulation had a skin penetration profile similar to that of a commercial formulation; however, the commercial formulation had twice the AZA concentration.^123^

**Figure 3:**
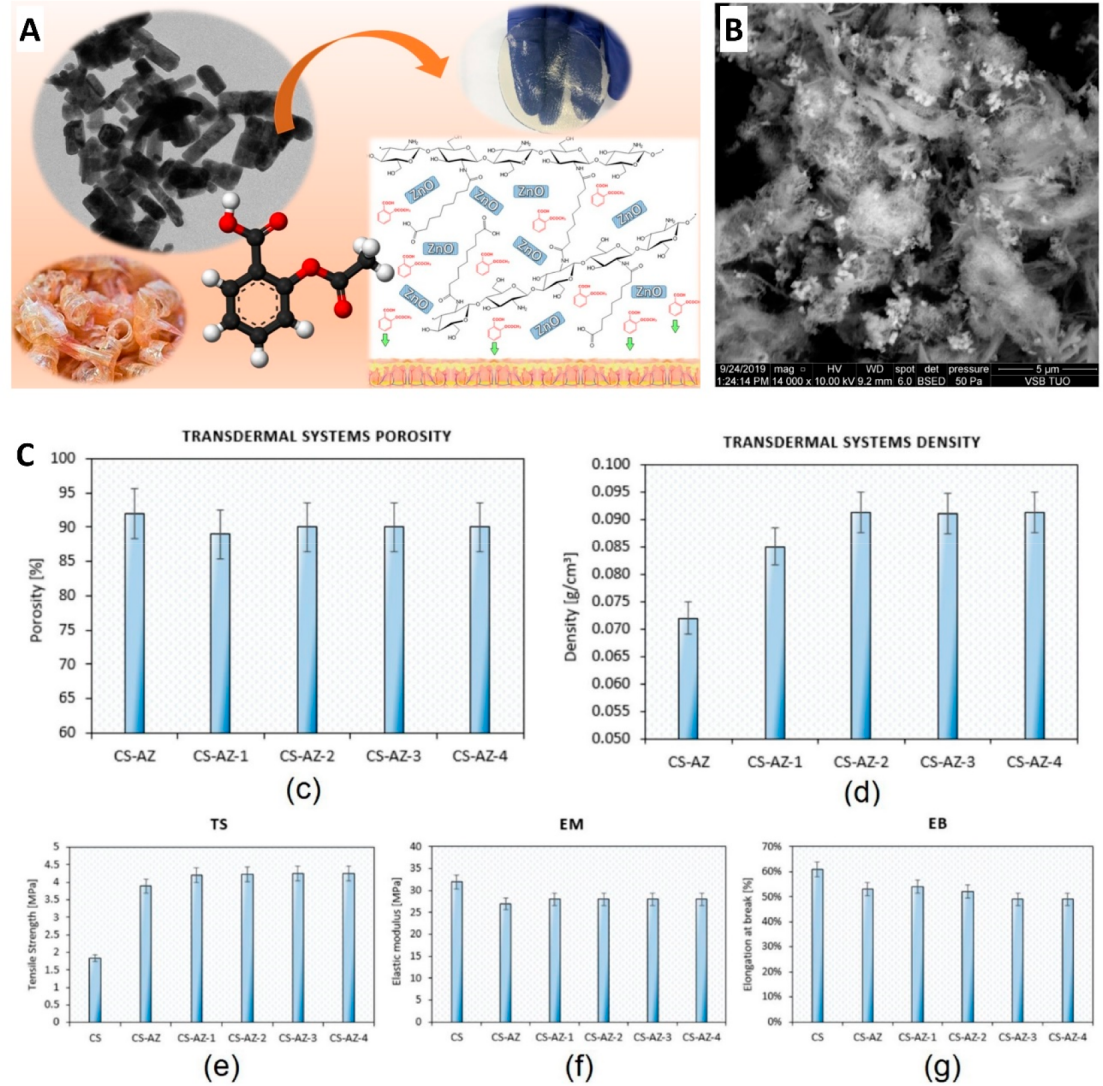
(A) Schematic representation of the prepared ZnO nanorods, with a transmission electron microscopy image. (B) Scanning electron microscopy image of the developed cross-linked hydrogel containing ZnO nanorods. (C) Characterization of the developed transdermal system, in terms of porosity, density, and mechanical properties (tensile strength, elastic modulus, and elongation at break). Adapted from Radwan-Praglowska et al.^124^ with permission from Elsevier. Copyright 2020 The Authors.

Radwan-Praglowska et al. proposed the formulation of ZnO nanorods functionalized with hydrogel cross-linked with AZA to obtain an effective system for cutaneous administration of AZA (Figure 3A and B).^124^ ZnO nanoparticles (NPs) have antibacterial properties, are stable and nontoxic, and increase the speed of delivery of the drug and the therapeutic efficacy, as their small size allows them to penetrate the epidermis and allows for a greater absorption capacity of the active substances due to their porous structure and large surface area.^124^ Chitosan is a biodegradable, biocompatible, nontoxic material that has a positive charge, so it increases the permeability of cell membranes and has a greater absorption capacity for the active substances due to its high porosity, but it cannot be used without being modified because it dissolves at pH values below 6.3 and the skin has a pH of 5.5; therefore, chitosan cross-linked with AZA prevents the degradation and rapid dissolution of the polymer at the skin pH. In this study, researchers cross-linked chitosan with AZA and posteriorly functionalized it with ZnO nanorods, intending to have a controlled release of the drug with long-term use.^124^ Chitosan cross-linked with AZA increased the porosity, and the higher the concentration of AZA, the greater the sorption capacity due to an increase in channels and carboxylic groups resulting from the cross-linking process (Figure 3C).^124^ The nanorods have a rectangular shape and are highly porous, which allows the incorporation of the drug and the controlled release of the drug, avoiding the burst effect.^124^ ZnO nanorods functionalized with hydrogels cross-linked with AZA present good water vapor permeability because the materials have higher porosity and an open-cell reticulated structure, and the decreased evaporation of water vapor can promote the controlled release of the drug, since water allows the accumulation of the drug in the polymer matrix and increases drug penetration into the skin due to the destabilization of well-organized structures.^124^ The hydrogel has several hydrophilic groups that have a good swelling capacity, which is important for drug release from the biomaterial, and has a high sorption capacity due to the presence of hydrophilic groups and to the open-cell porous structure, with this sorption capacity being important for penetration and the migration of water molecules in the three-dimensional matrix.^124^

Hanafi et al. proposed pretreatment with sonication of AZA–chitosan particles to decrease the particle size to 80 nm before applying electrospraying. Electrospraying allows droplets to be formed through the use of high-voltage fields and allows the drug to be encapsulated with high efficiency.^56^ In addition, it is a simple method that does not require the separation of the solvent particles and maintains the biological properties of the active components.^56^ In this study, the researchers initially prepared a solution of chitosan particles loaded with AZA through vigorous stirring for 60 min and subsequent dilution and sonication in continuous ultrasound at 400 W, which was then subjected to electrospray.^56^ The solution of chitosan particles loaded with AZA consisted of a mixture of AZA and chitosan at a 1:2 ratio at pH 5.0 ± 0,1.^56^ This solution was introduced into a 1 mL injection syringe and electrosprayed through a syringe pump with a flow of 1 mL/h and a voltage of 6.8 kV in a single conical jet mode with a distance between the nozzle and the collector of 1 cm.^56^ The morphology and particle size distributions of the nanoparticles obtained were studied using scanning electron microscopy, and the size and the size distribution were determined by dynamic light scattering (DLS).^56^ These nanoparticles showed a reduced size dispersion and an almost spherical shape.^56^ This study demonstrated that the chitosan–AZA solution yielded particles with smaller sizes, since sonication promotes the breaking of intra- and intermolecular bonds, with greater the amplitude and duration of sonication leading to smaller sizes of the particles.^56^

### 6.4 Timolol

Timolol is a nonselective β-blocker that causes vasoconstriction, induction of apoptosis, inhibition of angiogenic factors such as VEGF, and inhibition of inflammatory mediators such as MMP-2, MMP-9, and IL-6.^125^ This drug has a good cost-benefit ratio, good accessibility, low incidence of adverse reactions, and promotes a simple application.^125^

Mokadem et al. performed a multicenter study with patients who had ETR rosacea or PPR rosacea with the aim of studying the efficacy of topical timolol 0.5% in this population.^125^ The study lasted eight weeks, and the assessment of the severity of rosacea consisted of the IGA score and a clinical rosacea scale.^125^ The therapeutic regimen instituted for the study participants consisted of the application of 4–8 drops of topical 0.5% timolol to the affected areas of the face every night during the study period.^125^ Response to treatment was determined every two weeks until the end of the study and was based on the evaluation of rosacea through the degree of erythema, telangiectasias, papules, and pustules.^125^ The formulation used in the study proved to be safe since the adverse effects observed were referred to as mild and tolerable, and the use of emollients reduced these effects.^125^ The results obtained in the study suggest that timolol is more effective in ETR rosacea than in PPR rosacea, although no statistically significant improvements were obtained in any of the groups.^125^ Timolol mainly improved telangiectasia and erythema, with no significant effect on either papules or pustules, and prevented bacterial resistance.^125^ Thus, it is thought that the topical use of timolol may be beneficial in combination with other therapies, since most topical therapeutic options currently available act mainly on papules and pustules and do no have much effect in terms of telangiectasias and erythema, but more studies are needed to assess the benefit of this combination therapy.^125^

### 6.5 Ivermectin

Currently, topical ivermectin 1% cream offers an emerging treatment indicated for PPR rosacea.^16,25,126^ Considering that ivermectin is a macrocyclic lactone derivative, its therapeutic effect results from its anti-inflammatory activity, similar to other macrolides.^12^ Although the exact mechanism of action of ivermectin in treating inflammatory lesions of rosacea has not yet been clarified, its anti-inflammatory effects seem to result from the decrease in phagocytosis and neutrophil chemotaxis, inhibition of inflammatory cytokines, and negative regulation of TNF-α, LL-37, TRL4,^127^ IL-1B, and IL-10.^12,14,25,107^ Additionally, ivermectin is characterized by broad-spectrum antiparasitic action,^127,128^ presenting the ability to eliminate *Demodex mites*,^25^ a mite from pilosebaceous units of patients with PPR rosacea.^129^

In contrast to conventional topical treatment options, ivermectin 10 mg/g cream offers the advantage of a once-daily application.^130^ The main side effects of ivermectin are paresthesia, pruritus, and xerosis.^14,25^ The sustained decline^128^ in the number of inflammatory lesions, as well as the transient nature of the side effects, shows that ivermectin can be considered an effective and safe drug in patients with PPR rosacea.^14,128^

### 6.6 Calcineurin Inhibitors

Calcineurin inhibitors have anti-inflammatory action and decrease the activation of T cells, which in turn decreases the production and release of inflammatory mediators.^25^ The inhibition of proinflammatory mediators by this pharmacotherapeutic class makes it potentially effective in the treatment of rosacea PPR and ETR.^12,25^ The anti-inflammatory activity of this pharmacotherapeutic class led to the study of 0.1% tacrolimus ointment and 1% pimecrolimus cream.^12^ The topical application of these drugs showed a significant reduction in erythema,^12^ although it did not decrease the number of inflammatory lesions in patients with PPR rosacea.^12^ The use of these formulations does not cause telangiectasias or cutaneous atrophy,^107^ but they have a great potential for irritation^25^ and, since these drugs have immunosuppressive properties, they facilitate the development of *Demodex mites*.^107^ Thus, the use of these formulations should be restricted to patients whose clinical manifestations are resistant to other available treatments.^12^

### 6.7 Topical Retinoids

Topical retinoids promote the repair of photodamaged skin and negatively regulate the Toll2 receptor.^25,107^ They also have anti-inflammatory, free-radical scavenger, and keratolytic effects.^12^ Topical therapy with retinoids reduces erythema, telangiectasias, and the number of papules and pustules,^12,25^ but it may take two or more months until improvements are observed from its therapeutic action.^131^ However, topical retinoids usually cause skin irritation,^25^ so they are only used as an alternative treatment for individuals who have PPR rosacea.^12^

### 6.8 Permethrin

Permethrin is an antiparasitic drug^14^ that is marketed in the form of a 5% permethrin topical cream.^107^ The therapeutic use of this cream is mainly related to patients who have PPR rosacea,^107^ and its activity decreases erythema and papules but has no effect on telangiectasias, pustules, and rhinophyma.^12,14,25^ However, this approach proves to be powerful mainly due to the ability of permethrin to reduce the colonization of the skin by *Demodex folliculorum*.^107^

Ebneyamin et al. conducted a prospective, randomized, double-blind, placebo-controlled clinical trial for 12 weeks to assess the efficacy and safety of a gel formulation consisting of 2.5% permethrin with tea tree oil (TTO) in patients with rosacea PPR, and the effectiveness of the formulation was based on the detection of demodex density and clinical manifestation using standard skin surface biopsy (SSSB).^132^ The active constituent of the TTO tree is terpinen-4-ol, which has antiparasitic and anti-inflammatory activity, and this study evaluated the advantages of its inclusion in the formulation, as this oil has fewer side effects than other active constituents with this action.^132^ The composition of the study formulation consisted of carbomer 941, benzalkonium chloride, triethanolamine, TTO, and 99.88% purified permethrin. Participants in the study were asked to discontinue any treatment and/or cosmetics two weeks before study entry.^132^ The clinical trial protocol consisted of applying the test formulation on one side of the face and, after washing hands, applying the placebo on the other side of the face.^132^ The duration of the trial was 12 weeks, during which the participants applied the respective formulations twice a day, and the evaluation of demodex density was performed in weeks 2, 5, 8, and 12.^132^ The results of the study showed that after week 5 there was a reduction in demodex density on the side of the face treated with the study formulation, and the other side of the face had a decrease in density after 12 weeks of treatment, but this reduction was less than that obtained with the test formulation.^132^ At the end of the study, it was found that the formulation of permethrin with TTO showed a greater reduction in demodex density and greater therapeutic action in reducing erythema, papules, pustules, dry skin, and burning, although none of the formulations improved the plaques, telangiectasias, redness, and edema.^132^ The side effects observed were mild to moderate, thus showing that the formulation is safe.^132^

### 6.9 Serine Protease Inhibitor

Limón et al. proposed the formulation of a 4-(2-aminoethyl)benzenesulfonyl fluoride hydrochloride (AEBSF·HCl) supramolecular hydrogel, an irreversible serine protease inhibitor.^133^ The chosen gelling agent for hydrogel preparation was the bis imidazolium (1·2Br) using a 1:1 ratio of ethanol to water.^133^ The optimal hydrogel drug concentration was 5 mg/mL AEBSF·HCl, since it was the maximum drug concentration that allowed the rapid formation of the hydrogel, and the molar ratio of gelling agent to the drug was approximately 1:4.^133^ AEBSF·HCl was incorporated into the interstitial space and within the fibers; after the incorporation of AEBSF·HCl, the fibers were densely twisted much more than fibers without AEBSF·HCl.^133^ The researchers prepared hydrogels containing different concentrations of drugs to assess the influence of these concentrations on the morphology of the hydrogel during its storage.^133^ For this assessment, hydrogels were prepared and stored for two weeks in sealed vials.^133^ After this period, the researchers found that hydrogels containing 1 mg/mL AEBSF·HCl showed the same morphology as the fresh preparation, while hydrogels containing a concentration of AEBSF·HCl higher than 3 mg/mL presented coiled fibers due to the intermolecular interactions that occur between the cationic drug and gel nanostructure because a metastable state is formed during gelation, which through changes in external conditions can change the gel structure.^133^ In addition to the evaluation of the influence of drug concentration on the morphology of the hydrogel during its storage, after two weeks the researchers also evaluated the composition of the fibers and found that after the existence of drug within the fibers, as these fibers had sulfur in their composition, and the evaluation of the coiled fibers concluded that sulfur and fluorine were present in a lesser amount, suggesting that release of drug from fibers to interstitial space induces coiled fibers.^133^ The results obtained through differential scanning calorimetry (DSC) show that adding the drug to the gel makes it more stable at higher temperatures, since the gel starts to form at higher temperatures.^133^ When the AEBSF·HCl hydrogel is at higher temperatures, gelling occurs, and adsorption of the drug in the interstitial space of the gel to the fibers occurs as a result of an increase in the surface tension of the solvent.^133^ An *in vitro* drug release test showed that the hydrogel allows complete permeation of AEBSF·HCl to the skin, and during the first hours after application there is extensive drug delivery of the formulation to the skin.^133^ In addition to permeation and drug delivery, this study also showed that degradation of formulation at pH 5.5 (approximately the skin pH) is unlikely to occur.^133^

### 6.10 Pioglitazone

**Figure 4:**
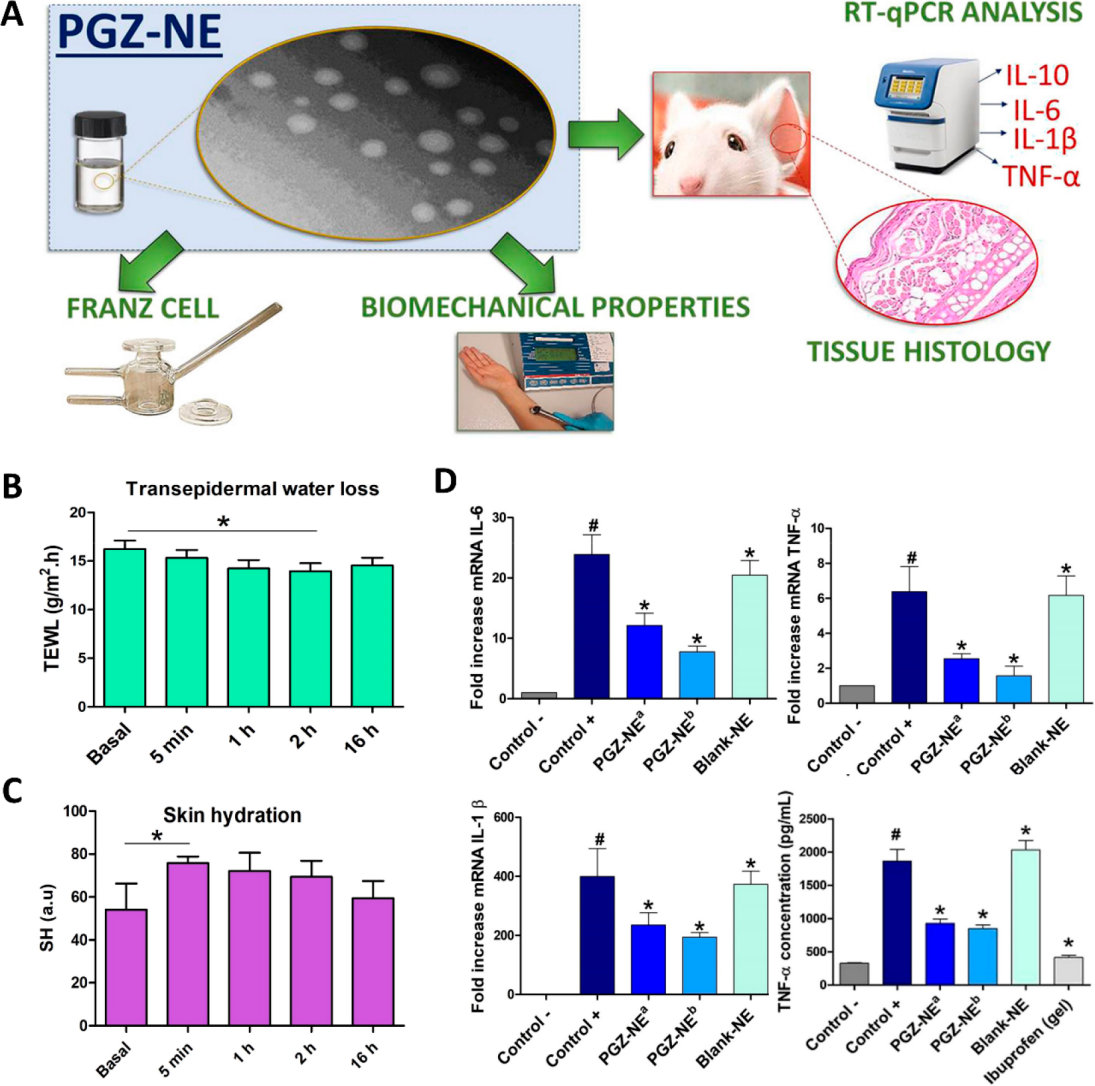
(A) Schematic representation of the developed PGZ NNE, including performed characterization assays. (B) Tolerance study results in humans involving TEWL measurement. (C) Tolerance study results in humans involving skin hydration measurements. (D) Relative expression of inflammatory cytokines IL-6, TNF-α, and IL-1β in mice. Adapted from Espinoza et al.^134^ with permission from Elsevier. Copyright 2019 Elsevier Inc.

Espinoza et al. proposed the formulation of a PGZ NNE for inflammatory dermatose treatment due to the ability of PGZ to decrease excessive production of proinflammatory cytokines or stop the inflammatory process (Figure 4A).^134^ PGZ is a class II drug, so the formulation consisted of an NNE to increase the solubility of PGZ in water to increase the therapeutic efficacy.^134^ For the NNE formulation, castor oil was used as the oil phase to which PGZ was added. Labrasol was used as a nonionic surfactant with a low potential to cause skin irritation, and ranscutol P and propylene glycol were used as cosurfactants because they are biocompatible with the skin. In addition, transcutol P is able to deposit intercellular lipids in the SC, and purified water was used as the aqueous phase.^134^ The final composition of the NNE is a PGZ concentration of 1 mg/mL, 6% castor oil, 52.9% labrasol, 9.87% transcutol P, 4.93% propylene glycol, and 26% purified water, as this formulation of the NNE presented itself as homogeneous, transparent, without precipitation, with a pH compatible with the skin (5.42), and the ability to deposit spherical nanodroplets on the skin. From stability studies, the NNE was stable for 60 days at 25 and 40 °C.^134^ After topical application of NNE, there was a TEWL decrease (Figure 4B) and an increase in the hydration of the SC (Figure 4C), confirming the biocompatibility of the NNE.^134^ The topical application of NNE decreased the production of TNF-α, IL-6, and IL-1β (Figure 4D) and decreased inflammatory cell infiltration, thus showing the anti-inflammatory effect of PGZ.^134^ PGZ release through the NNE shows a rapid release of PGZ at an early stage and subsequently a sustained release of PGZ, so NNE has proven to be an effective system for releasing PGZ into the skin; additionally, once the contact between the nanodroplets and the skin becomes more effective, greater cutaneous drug retention occurs, which increases the anti-inflammatory effect.^134^ The PGZ NNE presents Newtonian behavior allowing administration through a spray or roll-on form, since it has a high retention capacity and skin tolerability, thus promoting a long duration of action.^134^ the PGZ NNE showed great efficacy in this study, showing promise in its use for the treatment of rosacea.^134^

### 6.11 Dapsone

Dapsone is an anti-inflammatory and antimicrobial drug that is used for the treatment of inflammatory skin diseases.^135^ However, its low solubility in water restricts its incorporation into topical formulations.^135^ Thus, Elmowafy et al. studied the influence that the nanostructure of the lipid carrier has on the cutaneous yield of dapsone for the treatment of rosacea.^135^ Nanostructured lipid carriers (NLCs) were developed by the emulsification/sonication method, where the aqueous phase was prepared by mixing a 2% solution of different emulsifiers in double distilled water and the oil phase (10% w/v) was prepared using different proportions of solid lipids and liquid lipids using 0.5% lecithin as the lipophilic surfactant and dapsone.^135^ The proportion of solid lipid to liquid lipid had a significant influence on particle sizes, since the increase in this ratio resulted in larger particle size, influenced the viscosity, and influenced the encapsulated dapsone because the incorporation of dapsone in the lipid matrix depends on the incorporation of the liquid lipid in the solid lipid so that there is a change in the arrangement of the crystals to create space for drug entrapment.^135^ Thus, the greatest encapsulation efficiency was achieved when the NLCs were formulated with 7.5% labrafac lipophile and, in turn, 2,5% precirol ATO 5.^135^ The skin has a negative charge, so the deposition and distribution of dapsone in the skin are greater when the surface of the NLCs has a positive charge due to the electrostatic attraction between the negative charge of the skin and the positive charge of the lipid carrier.^135^ Furthermore, NLCs formulated with cetyltrimethylammonium bromide (CTAB) as a surfactant showed a slightly positive ζ-potential.^135^ Thus, the final composition of the NLCs was 2.5% precirol ATO, 7.5% labrafac lipophile, and 2% CTAB.^135^ This formulation showed a homogeneous particle size distribution, with no aggregation of rounded to elliptical particle shapes.^135^

*In vitro* release of dapsone from NLCs exhibits a biphasic mechanism where there is a burst effect at an early stage as a result of dapsone remaining on the NLC surface and, subsequently, a sustained release of dapsone due to the affinity between dapsone and the lipid matrix from NLCs, leaving a drug embedded deep in the lipid core so that its release from NLCs is prolonged.^135^ NLCs showed an occlusive effect *in vitro* because the solid lipids that make up the lipid matrix form a lipid film that prevents TEWL, thus improving skin hydration.^135^*Ex vivo* permeation studies confirmed the formation of this lipid film, which allows further penetration and diffusion of the dapsone through the skin by decreasing TEWL.^135^ On the other hand, drug penetration into the skin from NLCs is also related to the rearrangement of the lipid skin triggered by NLC application due to miscibility between NLC lipids and epidermal lipids.^135^ The drug diffusion from NLCs likewise relates to a higher surface area of the lipid carrier, which increases the contact area between the NCLs and the skin, and their small size also contributes to increased adhesiveness between the NLCs and the skin.^135^ Overall, these studies suggest that topical NCL application of dapsone, which presents a slightly positively charged ζ-potential, shows increased efficacy in the treatment of rosacea, as it reduced chemotaxis of neutrophils and did not provoke any skin irritation.^135^ Therefore, positively charged NLCs are a promising therapeutic approach for rosacea.^135^

### 6.12 Minocycline

Minocycline is a semisynthetic tetracycline derivative with anti-inflammatory, bacteriostatic, and antioxidant effects.^36^ FMX103 is a topical formulation in foam consisting of a minocycline micronized suspension^136,137^ whose excipients consist of soybean oil, coconut oil, light mineral oil, and cyclomethicone due to its compatibility with minocycline and the moisturizing effects of these ingredients.^136^ This formulation was developed for the treatment of moderate to severe PPR rosacea to increase therapeutic efficacy^136^ because of the better penetration into the skin of micronized minocycline,^137^ which reduces systemic exposure and its adverse effects.^136^ Jones et al. conducted a phase I, nonrandomized, open-label study to describe the pharmacokinetics (PK) of minocycline and to assess the safety of this formulation with a dosage of 2 g once daily for 14 days.^136^ PK determinations were performed by chromatographic separation using gradient conditions with tandem mass chromatography (LC–MS/MS) using plasma samples, and the safety assessment consisted of the treatment-emergent adverse effects (TEAE), the results of clinical laboratory tests, physical exams, and vital signs.^136^ Furthermore, IGA was used to assess erythema. Pharmacokinetic studies have demonstrated minimal systemic absorption of minocycline, with no evidence of drug accumulation, and a steady-state was achieved after the first application.^137^ The application of the formulation proved to be safe and well-tolerated, since there were no serious adverse effects.^137^

Mrowietz et al. conducted a randomized, double-blind, controlled phase II study to evaluate the safety, tolerability, and efficacy of FMX103 1.5% and FMX103 3% with daily application in the evening for 12 weeks.^138^ Study participants were divided into three groups in a 1:1:1 ratio, with one group receiving treatment with FMX103 1.5%, another receiving treatment with FMX103 3%, and another receiving treatment with a vehicle.^138^ The study of the effectiveness of these formulations was based on the count of inflammatory lesions and erythema evaluation through IGA and took into consideration the quality of life of the patients evaluated using the RosaQol questionnaire.^138^ The safety assessment consisted of physical examinations, vital signs, side effects, and clinical laboratory tests.^138^ In this study, FMX103 formulations showed a reduction in inflammatory lesions and improvements in higher IGA scores compared to the vehicle.^138^ The decrease in papules and pustules was statistically significant in the second week of the study in the groups that received treatment with FMX103, and the improvement of the IGA score in the two levels was verified from the fourth week of the study for the groups that received treatment with FMX103.^138^ Additionally, in the evaluation of erythema and quality of life, both formulations of FMX103 demonstrated superiority when compared to the group that received vehicle treatment.^138^ FMX103 formulations were found to be safe and well-tolerated, with no serious adverse effects related to treatment, and the severity of local signs and symptoms was similar in all study groups.^138^

**Figure 5:**
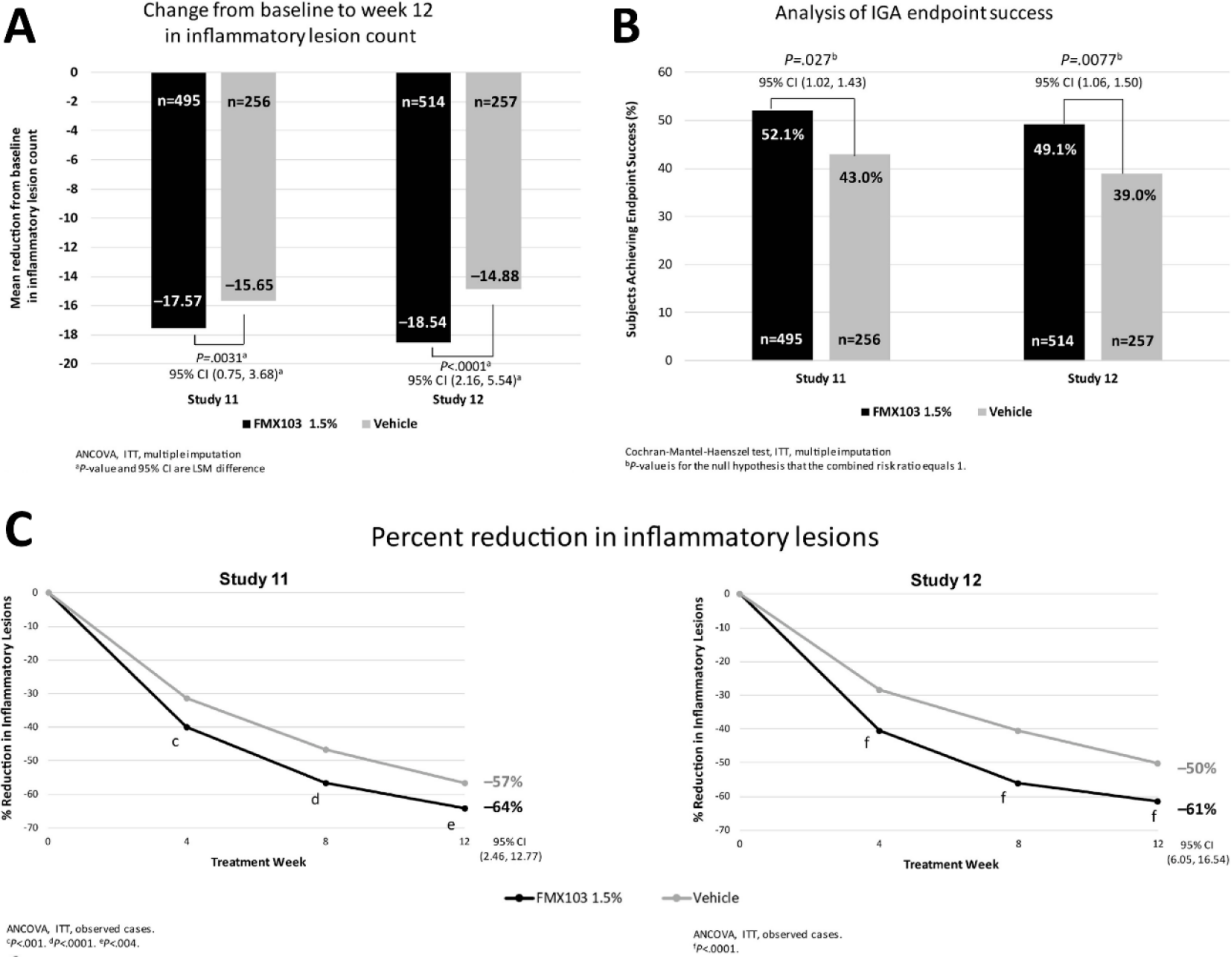
Efficacy end points after treatment regarding (A) inflammatory lesion count, (B) IGA end points, and (C) percent reduction in inflammatory lesions. Adapted from Gold et al.^137^ with permission from Elsevier. Copyright 2020 American Academy of Dermatology, Inc.

Gold et al. carried out randomized, double-blind, and vehicle-controlled phase II and III studies for 12 weeks. Study participants were divided into two groups in a 2:1 ratio, with one group receiving treatment with FMX103 1.5% and another receiving a vehicle.^137^ The treatment consisted of applying the respective formulation once a day in a thin layer.^137^ The study of the effectiveness of this formulation was based on the change in the number of inflammatory lesions and erythema evaluation through IGA, and the safety assessment consisted of physical examinations, vital signs, side effects, clinical laboratory tests, and the assessment of local tolerability.^137^ Patients who received treatment with the FMX103 formulation exhivited a statistically significant reduction in inflammatory lesions after the fourth week of treatment (Figure 5A and C) and greater improvement in the IGA (Figure 5B), showing the superior efficacy of the FMX103 formulation compared to the vehicle.^137^ In this study, as in the study by Mrowietz et al., the FMX103 formulation was shown to be safe and well-tolerated, with no serious adverse effects reported, and the most common adverse effects were mild problems such as burning, dryness, itching, flaking, or hyperpigmentation.^137^ Thus, this study showed greater effectiveness of the FMX103 1.5% formulation in relation to the control and revealed a greater adherence to therapy by the patients.^137^

## 7 Future Perspectives

### 7.1 Nanosystems as Recurring Tools for Topical Drug Delivery

Nanotechnology continues to be a sought-out approach to improve drug delivery, including in topical drug administration. Many types of nanosystems have been developed for topical drug administration, mainly for anti-inflammatory and anti-infectious purposes, not only to increase therapeutic efficacy but also as a weapon to fight antimicrobial resistance.^139−145^ The number of *in vitro* and *in vivo* studies regarding this topic recently multiplied, and clinical trials have also been performed evaluating the efficacy of a liposomal polyvinylpyrrolidone-iodine hydrogel for the treatment of not only rosacea but also several other types of dermatoses, such as acne vulgaris, atopic dermatitis, and impetigo contagiosa.^140^ Polyvinylpyrrolidone-iodine’s anti-inflammatory and antiseptic activity was added to the moisturizing, drug encapsulation, and controlled drug delivery capacity of liposomes, and results showed that the Global Clinical Severity score improved for all dermatoses after application of the developed liposomal hydrogel for 4 weeks. There were improvements not only in pain and eczema area but also overall quality of life, and the treatment was well tolerated by participants, with only mild side effects being reported, such as burning or itching sensations. Thus, the developed liposomal hydrogel showed promising results in the treatment of several inflammatory skin conditions associated with infection.

Another relevant study on this field is the one by Wang et al.,^146^ in which the authors developed several types of phospholipid-based nanovesicles, namely liposomes, hexosomes, glycerosomes, and ethosomes, loaded with tretinoin for the treatment of rosacea. All developed nanocarriers showed small and homogeneous particle sizes (60–132 nm, with a polydispersity index between 0.23 and 0.29), with spherical and negatively charged particles (ζ-potential between −19 and −29 mV) and variable entrapment efficiency (32–63%). Additionally, *in vitro* and *in vivo* studies showed that all developed nanocarriers promoted drug deposition in the skin’s *stratum corneum* with reduced permeation to the systemic circulation, which is ideal for formulations that are meant to act on the upmost layers of the skin. As for therapeutic efficacy, the developed vesicles led to a marked attenuation of edema and inflammatory cells in an *in vivo* croton oil-induced skin model. Hence, the developed tretinoin nanosystems have proven to have potential efficacy in the treatment of rosacea, with this being only one example of the great potential of nanosystems for the treatment of skin diseases.

### 7.2 Probiotics

According to the FDA, probiotics are “live microorganisms that are intended to have health benefits when consumed or applied to the body”. Rosacea is a chronic inflammatory dermatosis^11^ with differences in the skin microbiome^147^ that functions as a protective barrier.^147^ Several studies have been carried out, and a relationship has been observed between some microorganisms, such as *Demodex mites*, *Helicobacter pillory*, and *Staphylococcus epidermidis*, among others, and rosacea.^148^ The studies suggest that this may be the result of several factors: age, since a lower relative abundance of *C. acnes* was observed in older Caucasian patients; the severity of rosacea; skin temperature, since patients who have rosacea have a higher temperature that causes *S. epidermidis* to show β-hemolytic activity; gender; pH; and ethnicity, among others. In addition, *Demodex mites* also contribute to inflammation in rosacea, since chitin that is present in the composition of these mites can trigger a proinflammatory response of keratinocytes through TLR-2 and increase the production of cathelicidin, TNF-α, and IL-8 triggered by *B. oleronius* antigens. *S. epidermidis* antigens also play an important role in skin inflammation, as they are recognized by TRL-2.^11^ Studies of the microbiome of the skin have focused on the topical application of probiotics to restore the cutaneous microbiome and, consequently, contribute to immunological homeostasis.^147^ These studies with topical probiotics had discrepant results, which may be associated with bacterial species, as each species has unique characteristics.^147^ Rosacea patients may have gastrointestinal comorbidities, suggesting that there may be a relationship between rosacea and the intestinal microbiota, although the association between rosacea and the intestinal microbiome is not yet fully understood.^11^ Aleh et al. conducted a study with patients who tested positive for the fecal antigen test of *Helicobacter pylori*.^149^ The established therapy for these patients consisted of 500 mg of MTZ twice daily, 500 mg of clarithromycin twice daily and 40 mg of pantoprazole once a day, and these patients were followed for one year.^149^ In this study, there was a significant reduction in the intensity of clinical signs and symptoms of rosacea, except for the phymatous changes and telangiectasias, which may be associated with the longer period of time that is necessary for these two clinical signs to show a decrease, with a greater improvement in patients with PPR rosacea than in patients with ETR rosacea.^149^ However, these results are not clear, since this study did not have a control group.^149^

Topical probiotics are promising in the treatment of rosacea; however, more studies are needed, especially concerning their safety.^147^ Furthermore, the mechanism of action of probiotics is not yet fully understood, and the anti-inflammatory effect through the stimulation of T cells has been proposed.^147^ Another factor that also affects the topical use of probiotics is the regulatory gaps of the FDA regarding the approval and classification of probiotics.^147^

### 7.3 Anti-TNFα and siRNA

**Figure 6:**
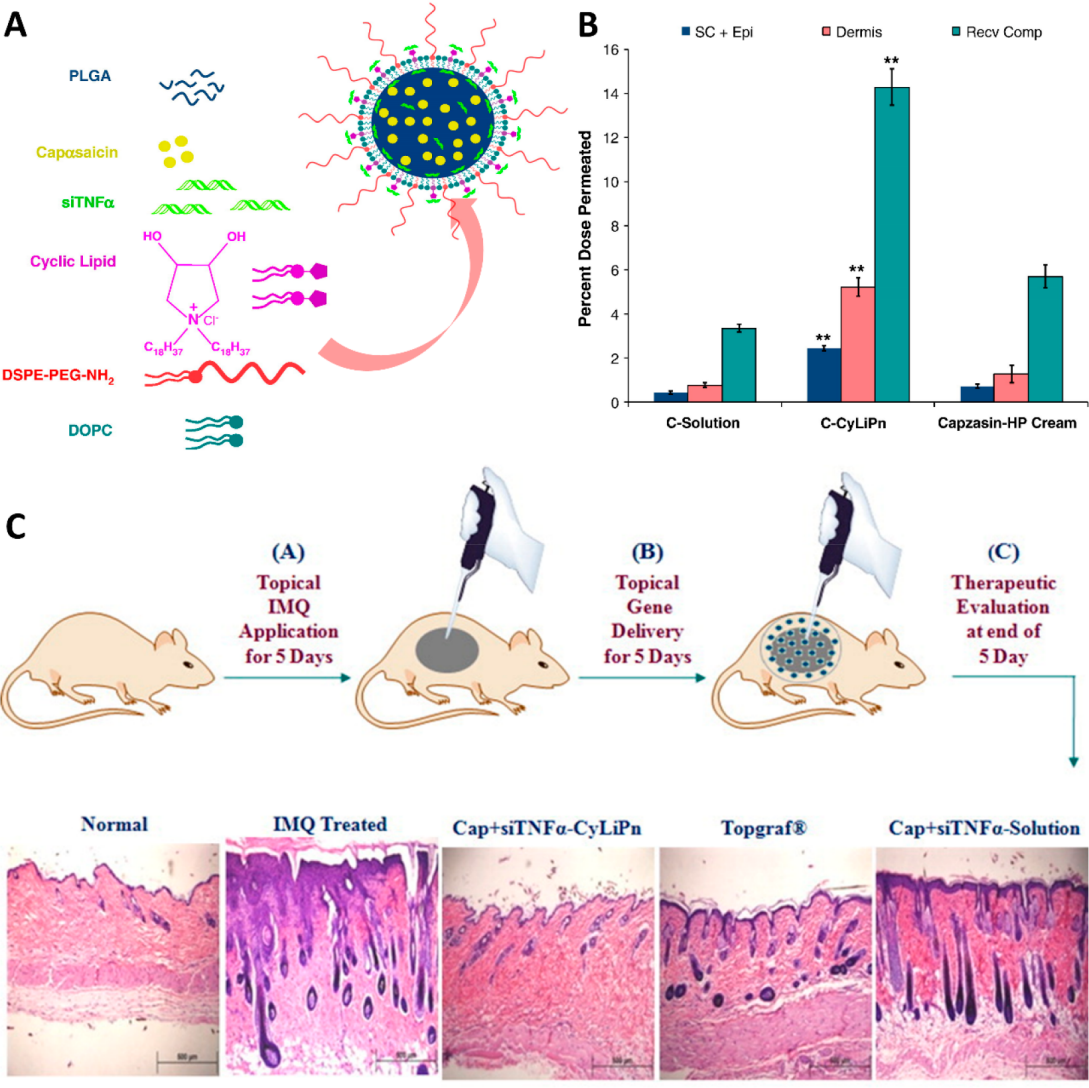
(A) Schematic representation of the developed hybrid lipid–polymer nanocarriers, with respective composition. (B) *In vitro* rat skin permeation results. (C) *In vivo* assay representation, with hematoxylin and eosin histological staining images. Adapted from Desai et al.^150^ with permission from Elsevier. Copyright 2013 Elsevier B.V.

Rosacea is characterized by an increase in the levels of inflammatory mediators such as TNF-α. siRNAs are small interference RNAs of proinflammatory cytokines at the mRNA level and constitute a therapeutic approach to chronic inflammatory diseases.^150^ Thus, Desai et al. proposed the formulation of hybrid lipid–polymer nanocarriers with a cationic head (CyLiPn) of capsaicin (Cap) and anti-TNFα siRNA (siTNFα) (Figure 6A) and the evaluation of their therapeutic efficacy in the treatment of chronic inflammatory diseases.^150^ These nanocarriers allow for coadministration of the drug, Cap, and siRNA-based therapy.^150^ Cap is an anti-inflammatory drug that inhibits the production of PGE_2_ and stimulates the release of vasoactive neuropeptides and calcitonin gene-release peptide (CGRP).^150^ The CyLiPn formulation consisted of a PLGA layer that presents a negative charge and that constituted the hydrophobic polymer core in which Cap was encapsulated, since it is a poorly water-soluble drug; an outer layer of 1,2-dioleoyl-*sn*-glycero-3-phosphocholine (DOPC);1,2-distearoyl-*sn*-glycero-3-phosphoethanolamine-*N*-[amino(polyethylene glycol)-2000] (ammonium salt) (DSPE-PEG2000), which is a hydrophilic polymer that forms the stealth shell of NP; and a lipid monolayer formed by a cationic lipid formed by the cyclic pyrrolidinium headgroup at the interface of the hydrophobic core and hydrophilic shell to deliver siRNA into deeper layers of the skin.^150^ This formulation showed a homogeneous size distribution (approximately 160 nm) and had a positive ζ-potential, which is related to the quaternary ammonium in the pyrrolidinium headgroup of the cationic lipid having a positive charge, a desirable characteristic to protect the siRNA from a negative charge of degradation and to maximize transfection efficiency.^150^ The presence of pyrrolidone in the lipid monolayers increases the transdermal absorption of the formulation due to the increased fluidity of the lipid layer in the liposome.^150^ CyLiPn is potentially safe and biocompatible, since PLGA and PEG-DSPE have been approved by the FDA for medical applications, and the presence of a cyclic head lipid can deliver siRNA and drugs to the deep layers of the skin and at therapeutic levels. *In vitro* studies showed that CyLiPn is potentially noncytotoxic, allows for greater drug release than the commercial formulation, and has a sustained drug release that results from the cationic surface of NP as well as the nanocarrier.^150^*In vivo* studies (Figure 6C) were performed using an imiquimod-induced psoriatic plaque-like model, which was used as a model of chronic skin inflammation.^150^ Topical application of CyLiPn resulted in a greater anti-inflammatory response due to increased skin permeation and was also related to the synergistic effect between Cap and TNFα.^150^ The increase in skin permeation results (Figure 6B) from the interaction between the SC and CyLiPn in which, in an initial phase, there is an ionic interaction between SC proteins and lipids that have a negative charge and the negative charge of CyLiPn, which can lead to the destabilization of the membrane when the threshold concentration of CyLiPn is exceeded.^150^ Thereafter, CyLiPn forms a film in the SC, which causes greater hydration of the SC and promotes greater penetration of CyLiPn. Finally, CyLiPn and the encapsulated substances can penetrate through the hair follicles, which may have a reservoir effect.^150^ This study shows that CyLiPn may be a promising therapeutic approach in the treatment of rosacea.^150^

### 7.4 Quality by Design

The development of nanocarriers as carriers of therapeutic molecules and/or cosmetic ingredients is a promising approach because of their small size, which enables them to increase skin permeation; this makes it easier to reach the specific target at appropriate concentrations and thus promotes greater safety and effectiveness.^151^ In addition to these benefits, nanocarriers also improve the solubility of active substances or cosmetic ingredients as well as their bioavailability. Despite these benefits, the production and use of nanocarriers have some limitations, since the manufacturing process has limited reproducibility and is complex and expensive. Additionally, in most cases the knowledge of the manufacturing process is limited by what is crucial the implementation of approaches to optimize and increase knowledge about them. QbD is a scientific and systematic approach that allows increasing knowledge of the entire process inherent to the nanosystem, thus allowing robust manufacturing processes, high-quality products, and real-time quality control with subsequent release in real-time, and at a regulatory level it has greater flexibility and less regulatory burden, as it grants greater scientific knowledge about the entire process. QbD starts with predefined objectives and emphasizes the importance of knowledge of the product, process, and control based on solid science and quality risk management. The implementation of this approach requires defining the quality target product profile (QTTP) and the critical quality attributes (CQAs) of the product, performing a risk assessment to identify critical process parameters (CPPs) and critical material attributes (CMAs), defining the design space through design experiments (DoE), and establishing a control strategy and continuous improvement and innovation throughout the product’s life cycle. Thus, at an initial stage, all the variables that may have an impact on the quality of the product are identified, although the impact of each of these variables may be different according to the quality attribute. After identifying all these variables, the critical formulation parameters are selected to proceed with optimization through factorial planning (DoE).^151^ DoE, as a structured and organized experimental process, allows, in a first phase, the evaluation of several parameters simultaneously in a reduced number of tests to identify CMA and CPP; after identifying CMA and CPP, an optimization phase follows where these are analyzed variables to identify the optimal conditions of these variables to avoid failure in the product’s CQA, which, in turn, guarantees its QTTP. The formulation of nanocarriers is complex, since their therapeutic efficacy, quality, and stability depend on numerous factors, and scale transposition is not always achieved. Thus, it is essential to implement a robust system that allows increasing knowledge of materials, formulation, and process parameters so that it is possible to optimize production methods and recognize possible problems of scale transposition. In this sense, QbD is a promising approach to obtain nanoformulations.^151^

### 7.5 Skin Color

Rosacea is an inflammatory dermatosis that is classified into four subtypes according to the clinical signs present.^152^ This condition was for many years considered a dermatosis of fair-skinned people, especially Fitzpatrick I and II phototypes; this idea is dated, as this pathology also affects people of colored skin.^152^ Rosacea is often underreported in people with darker phototypes due to the difficulty in recognizing clinical signs in phototypes with more pigmented skin, so that, in many cases, the diagnosis is made late.^46,152^ Thus, since erythema and telangiectasias are difficult to observe in these patients,^46,153^ their diagnosis should focus on other clinical signs, such as edema, dry appearance, thickening of the skin, and facial papules and pustules.^46^ Diova et al. performed a retrospective review with the aim of better understanding the clinical aspects of rosacea in people with Fitzpatrick phototypes V and VI.^153^ Through this review, they found that the most common clinical signs in phototype V consisted of erythema, telangiectasias, and erythematous papules and that the most common clinical signs in patients with phototype VI were predominantly skin-colored papules without pustules or telangiectasias, and some patients still had phymatous lesions.^153^ In this population, the subtype of rosacea that is most frequently diagnosed is granulomatous rosacea, which can be in part explained by late diagnosis due to incorrect diagnoses of another dermatosis, such as systemic lupus erythematosus, seborrheic dermatitis, and dermatomyositis, among others^5^ resulting from inconspicuous clinical signs of rosacea.^5,46,153^ Thus, in the clinical approach to these patients, specific techniques must be used to obtain a correct diagnosis.^5,153^ In this sense, some approaches that can be used include skin whitening to verify the presence of erythema and telangiectasias, photographing the affected areas against a blue background, and verifying the distribution, morphology, and color of papules and pustules, and adequate lighting should also be used to facilitate the observation of telangiectasias.^5,153^ The therapeutic approaches instituted for this population are the same as those applied to fair-skinned individuals, although there are little data on their safety and efficacy and, due to the existing differences between fair-skinned and colored skin, several factors must be taken into account when instituting therapy in people with colored skin, for example, changes in skin pigmentation.^46^

Therefore, it is important to carry out more studies in this population to increase knowledge regarding the clinical characteristics of rosacea in these individuals so that the diagnosis is made early, and it is also important to increase knowledge about the safety and efficacy of the treatments instituted as well as to determine the side effects that can result from them.^5^

### 7.6 New Diagnostic Devices

The diagnosis of rosacea is not always easy, especially in people with colored skin, because the morphological characteristics of the lesions can be confused with other dermatological diseases.^24^ The diagnostic procedures currently used are expensive, time-consuming, and often invasive, causing discomfort to the patient.^24^ Therefore, the development of new diagnostic devices is crucial.^24^ Thermal sensing is a new noninvasive technique that allows skin properties to be obtained as a function of depth up to several millimeters.^24^ The skin hydration sensor (SHS) is a noninvasive device that adheres to the body surface and allows us to obtain the local volumetric mean values of water in the skin under study, and this method has high robustness, precision, and reliability.^24^ In addition to these advantages, the removal of SHS does not cause irritation or damage to the skin, which is extremely important and can be used outside of clinical and/or experimental environments.^24^ This sensor is useful not only for diagnosing skin diseases but also for evaluating the therapeutic effectiveness of prescribed treatments.^24^ Thus, the SHS is a promising device for diagnosing and monitoring skin diseases.^24^

### 7.7 Theranostic Role of Mast Cells

Mast cells (MCs) are hematopoietic lineage cells present in virtually all body tissues and are part of the innate immune system.^24^ Recently, some studies have shown that patients with rosacea have a higher number and activity of MCs in the skin^24,24^ (detected by increased expression of chymase and MM9 mRNA^24^) when compared to individuals without this pathology^24^ and, in patients with ETR and PPR, the number of MCs is significantly higher in the lesions than in the uninvolved skin, with a positive correlation between the density of MCs and the duration of rosacea.^24^ These data suggest that MCs may play an important role in the pathogenesis of rosacea, as their activation leads to the release of pro-inflammatory cytokines, chemokines, proteases, antimicrobial peptides,^24^ and different growth factors.^24^ MCs, as innate immune cells, participate in the defense of the organism.^24^ In the presence of LL-37, degranulation of MCs can occur, which in turn releases pro-inflammatory cytokines such as IL-6 and MMP9 that potentiate inflammation, facilitating the development of rosacea through an innate immune response.^24^ Stress also promotes the degranulation of MCs with consequent release of histamine, tryptase, and other mediators that jointly promote inflammation, causing redness, itching, erythema, and/or burning sensation.^24^ In addition to these symptoms, the release of histamine and tryptase by MCs enhances MMP activity, which may lead to an increase in the fibrillar extracellular matrix, culminating in a state of fibrosis.^24^ In the degranulation process, MCs also release pro-angiogenic molecules such as VEGF, histamine, and tryptase that directly stimulate the migration and/or proliferation of endothelial cells, facilitating vascularization and angiogenesis; additionhistamine can cause vasodilation and increased vascular permeability, resulting in erythema and flushing.^24^ MCs, as cells of the innate immune system, initiate the recruitment of neutrophils, which in turn promotes increased permeability of blood vessels and the release of chemokines.^24^ These data suggest that MC-stabilizing drugs may be a new therapeutic target for the treatment of rosacea.^24^ In this sense, tests were carried out with cromolyn sodium, hydroxychloroquine, and artemisinin.^24^ Cromolyn sodium is a drug that inhibits the degranulation of MCs and, consequently, inhibits the release of inflammatory mediators and decreases MMP activity.^24^ Although there are no published data on the efficacy of this drug in the treatment of rosacea, the application of topical cromolyn sodium 4% cream reduces the itching associated with atopic dermatitis, thus being a promising drug for the treatment of rosacea, although specific studies are needed in regard to rosacea.^24^ Hydroxychloroquine is an antimalarial drug that has been shown to have anti-inflammatory efficacy when used in the treatment of dermatoses.^24^ Li et al. used animal models to study the role and mechanism of this drug when used in the treatment of rosacea.^24^ Through this study, they concluded that hydroxychloroquine decreases the activity of MMP and MC tryptase, which consequently reduces the activation of MCs.^24^ Wang et al. conducted a multicenter, randomized, double-blind pilot study to investigate the efficacy and safety of this drug in the treatment of rosacea.^24^ This study revealed that this therapy improves the quality of life of people with rosacea and causes few adverse effects.^24^ These results suggest that hydroxychloroquine may be a promising drug in the treatment of rosacea; however, further studies involving a larger sample size are needed.^24^ Artemisinin, like hydroxychloroquine, is an antimalarial drug that has anti-inflammatory and antiangiogenic properties.^24^ Yuan et al. carried out a study in an animal model of rosacea to evaluate the role that this drug could have in the treatment of this dermatosis.^24^ In the animal model, this drug suppressed LL37-induced MC activation and, when applied to human endothelial cells, reduced angiogenesis.^24^ Brimonidine gel is a well-established therapeutic strategy for the treatment of rosacea, as it reduces facial erythema due to vasoconstriction of superficial vessels.^24^ Recently, studies carried out in animal models of rosacea showed that this gel, in addition to the vasoconstrictor effect, also decreases the mRNA levels of MC-specific enzymes increased by LL37 and, consequently, inhibits MC-induced inflammation.^24^ Botulinum toxin has recently emerged as a treatment for the persistent erythema and flushing associated with rosacea, although its mechanism of action is not fully understood.^24^ Recently, preliminary studies were performed that showed that botulinum toxin exerts a direct inhibitory effect on MCs, as it blocks MC degranulation by cleaving SNARE proteins within the cell.^24^

## 8 Conclusion

**Figure 7:**
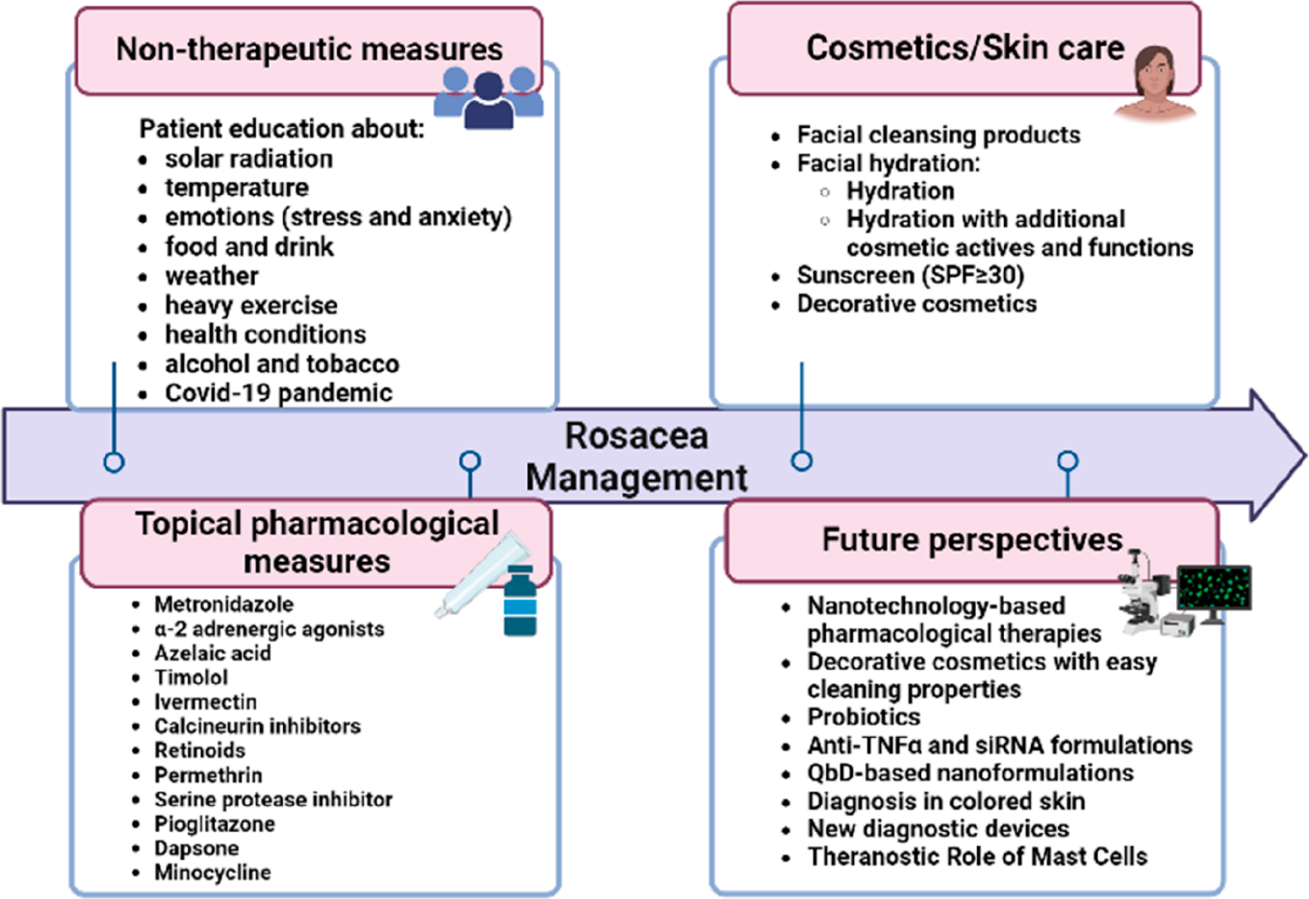
Representative rosacea management scheme: nontherapeutic measures, cosmetics/skin care, topical pharmacological measures, and future challenges (produced with Biorender).

Rosacea is a chronic inflammatory dermatosis with a negative impact on the quality of life and self-esteem of patients. It is essential to educate people to avoid triggering factors and to have greater control in the management of the disease. The pathological mechanisms of the disease are not yet fully understood, although it is known that this disease is multifactorial. This Review focuses on currently available topical treatments and cosmetic care, as well as new knowledge that has emerged regarding topically applied products (Figure 7). Advances in the production technology of pharmaceutical and cosmetic forms over the years have had a significant impact on addressing this pathology because they allow the market of various dosage forms to meet the needs and consumer preferences. In this context, encapsulated products such as 5% encapsulated benzoyl peroxide (E-BPO) cream (Epsolay; Sol–Gel Technologies Ltd.), and Twyneo (Sol–Gel Technologies Ltd.), a combination of microencapsulated tretinoin 0.1% and microencapsulated BPO 3% cream, approved by the FDA in mid-2021 provided treatment options with enhanced tolerability thanks to their controlled release of encapsulated material. Therefore, advances in pharmaceutical nanotechnology have a promising role in the treatment of dermatological conditions because they allow smaller particle sizes to be obtained, which in turn more easily penetrate the skin barrier, resulting in greater therapeutic efficacy and consequently increasing the quality of life of patients.

## Abbreviations

ACD: Allergic contact dermatitis
AEBSF. HCL: 4-(2-Aminoethyl)-benzenesulfonyl fluoride hydrochloride
AMP: Antimicrobial peptide
AZA: Azelaic acid
BoNT: Botulinum toxin
Cap: Capsaicin
CGRP: Calcitonin gene-release peptide
CMAs: Critical material attributes
CNP: Single-nucleotide polymorphism
CPPs: Critical process parameters
CQAs: Critical quality attributes
CTAB: Cetyltrimethylammonium bromide
CyLiPn: Hybrid lipid–polymer nanocarriers with cationic heads
DLS: Dynamic light scattering
DoE: Design experiments
DOPC: 1,2-Dioleoyl-*sn*-glycero-3-phosphocholine
DSC: Differential scanning calorimetry
DSPE-PEG2000: 1,2-Distearoyl-*sn*-glycero-3-phosphoethanolamine-*N*-[amino(polyethylene glycol)2000]
EDDP: Erythema-directed digital photography
ETR: Erythematotelangiectatic rosacea
FDA: Food and Drug Administration
SPF: Solar protection factor
HLA: Human leukocyte antigen
IDR: Intrinsic dissolution rate
IGA: Investigatoŕs Global Assessment
IL: Interleukin
LC–MSMS/MS: Chromatographic separation using gradient conditions with tandem mass chromatography
LMWG: Low-molecular-weight gelator
LNS-AZA: Lyophilization of an AZA nanocrystal suspension
LNS-AZA-PHA: Pluronic F127/hyaluronic acid hydrogels loaded with a lyophilizate of an azelaic acid nanocrystal suspension
MCs: Mast cells
MIC: Minimum inhibitory concentration
MTZ: Metronidazole
NF-KB: Nuclear factor-κB
NLC: Nanostructured lipid carrier
NNE: Nanoemulsion
NP: Nanoparticle
PGE_2_: Prostaglandin E2
PGZ: Pioglitazone
PK: Pharmacokinetics
PPR: Papulopustular
QbD: Quality by design
QTTP: Quality target product profile
ROS: Reactive oxygen species
ROSCO: Rosacea consensus
SC: Stratum corneum
SHS: Skin hydration sensor
SLS: Sodium lauryl sulfate
Smix: Mixture of surfactant and cosurfactant in a proportion (w/w) of 2:1
SNP: Single-nucleotide polymorphism
SSSB: Standard skin-surface biopsy
TEAE: Treatment-emergent adverse effects
TEWL: Transepidermal water loss
*T*_gel_: Sol–gel transition temperature
Th: T helper
TLR: Toll-like receptors
TNF-α: Tumor necrosis factor-α
TTO: Tea tree oil
UV: Ultraviolet
